# SHIP: a computational framework for simulating and validating novel technologies in hardware spiking neural networks

**DOI:** 10.3389/fnins.2023.1270090

**Published:** 2024-01-08

**Authors:** Emanuele Gemo, Sabina Spiga, Stefano Brivio

**Affiliations:** CNR–IMM, Unit of Agrate Brianza, Agrate Brianza, Italy

**Keywords:** spiking neural network, compact model, temporal progress, data flow, simulation platforms, supervised training, neuromorphic engineering

## Abstract

Investigations in the field of spiking neural networks (SNNs) encompass diverse, yet overlapping, scientific disciplines. Examples range from purely neuroscientific investigations, researches on computational aspects of neuroscience, or applicative-oriented studies aiming to improve SNNs performance or to develop artificial hardware counterparts. However, the simulation of SNNs is a complex task that can not be adequately addressed with a single platform applicable to all scenarios. The optimization of a simulation environment to meet specific metrics often entails compromises in other aspects. This computational challenge has led to an apparent dichotomy of approaches, with model-driven algorithms dedicated to the detailed simulation of biological networks, and data-driven algorithms designed for efficient processing of large input datasets. Nevertheless, material scientists, device physicists, and neuromorphic engineers who develop new technologies for spiking neuromorphic hardware solutions would find benefit in a simulation environment that borrows aspects from both approaches, thus facilitating modeling, analysis, and training of prospective SNN systems. This manuscript explores the numerical challenges deriving from the simulation of spiking neural networks, and introduces SHIP, Spiking (neural network) Hardware In PyTorch, a numerical tool that supports the investigation and/or validation of materials, devices, small circuit blocks within SNN architectures. SHIP facilitates the algorithmic definition of the models for the components of a network, the monitoring of states and output of the modeled systems, and the training of the synaptic weights of the network, by way of user-defined unsupervised learning rules or supervised training techniques derived from conventional machine learning. SHIP offers a valuable tool for researchers and developers in the field of hardware-based spiking neural networks, enabling efficient simulation and validation of novel technologies.

## 1 Introduction

Spiking Neural Networks (SNNs) have seen a rapid surge in interest in recent years (Indiveri et al., 2011; Bouvier et al., 2019; Young et al., 2019; Brivio et al., 2022; Christensen et al., 2022; Hendy and Merkel, 2022; Yamazaki et al., 2022), due to their potential reduction of the energy cost of conventional computing paradigms (Roy et al., 2019; Christensen et al., 2022). SNNs rely on the binary spike temporal encoding of the information transfer and processing, which can be ideally approximated as a cascade of information-dense events, sparsely taking place across the temporal coordinate. This indeed limits the energy flux into the SNN system. Additionally, in contrast to conventional (digital) processing units, SNN hardware realizations may carry out part of the computational tasks exploiting passively-evolving physical phenomena, which also lowers the need for a constant energy supply. For instance, neuronal or synaptic circuitry can use the discharge of capacitors (see e.g., Hwang et al., 2022), or the dissolution of the conduction path in a memristor (see e.g., Covi et al., 2016; Brivio et al., 2022), to implement the required temporal dynamics. Both the fundamentally-efficient processing paradigm and the contextually-efficient analog neuromorphic hardware drive the research toward novel hardware solutions.

As in most cutting-edge studies, any costly fabrication step would likely follow a thorough simulation study, that estimates the SNN potential performance metrics (Vineyard et al., 2019). The simulation of SNN systems may exploit dedicated hardware processors or accelerators, such as SpiNNaker (Mayr et al., 2019), Neurogrid (Benjamin et al., 2014), Truenorth (Akopyan et al., 2015), BrainScaleS (Pehle et al., 2022), Loihi (Davies et al., 2018), the DYNAPs family (Moradi et al., 2018), and Tianjic (Pei et al., 2019). However, these tools do not often provide the needed flexibility to include models of novel hardware technologies and functional blocks, possibly including beyond-CMOS concepts such as memristive elements. This limits the simulation methodology to algorithmic solutions.

FEM or SPICE-like simulations provide physically-realistic responses, but they are often applicable only to size-limited systems. Instead, assemblies of compact models can simulate complex systems and deliver relatively accurate simulation results, without necessarily implying a massive computational requirement. Nevertheless, the computational cost of simulating SNN systems remains a major challenge, as software tools can not take advantage of the continuous, parallel, event-driven processing that is exclusive to hardware systems.

In our initial review, we counted at least 37 actively maintained or stable platforms that support the simulation of SNN systems, each one dedicated to a narrow audience of neuroscience investigators or data-scientists. This suggests that orienting oneself towards the most suitable choice can be a tedious task. Howerver, we note that few of them are intended for the study of novel technologies for hardware systems, explicitly combining (i) a naturally-understandable coding framework, (ii) tools to analyze the behavior of the simulated systems, and (iii) data-oriented routines to test the potential performance of the simulated systems. It is our opinion that a tool combining the mentioned features would be of great benefit to neuromorphic engineers to rapidly prototype potential SNN systems, without incurring a steep learning curve.

For this reason, we devised a simulation platform, SHIP (*Spiking (neural network) Hardware In PyTorch*), developed to support the conception, design, investigation, and validation of novel technologies (as materials, devices, or circuits) in SNN architectures. The user of SHIP seeks a simple tool to rapidly simulate potential hardware SNN realizations, without mandating the prerequisite knowledge of the SNN simulation theoretical elements. To meet these objectives, SHIP helps towards (i) the definition of neuronal/synaptic circuitry models, (ii) the network simulation and behavioral analysis, and (iii) network performance assessment after rapid synaptic weight training. We anticipate that, as we develop SHIP in Python (a heavily object-oriented language), we exploit bespoke classes to interface the user with the platform algorithms.

**Figure 1:**
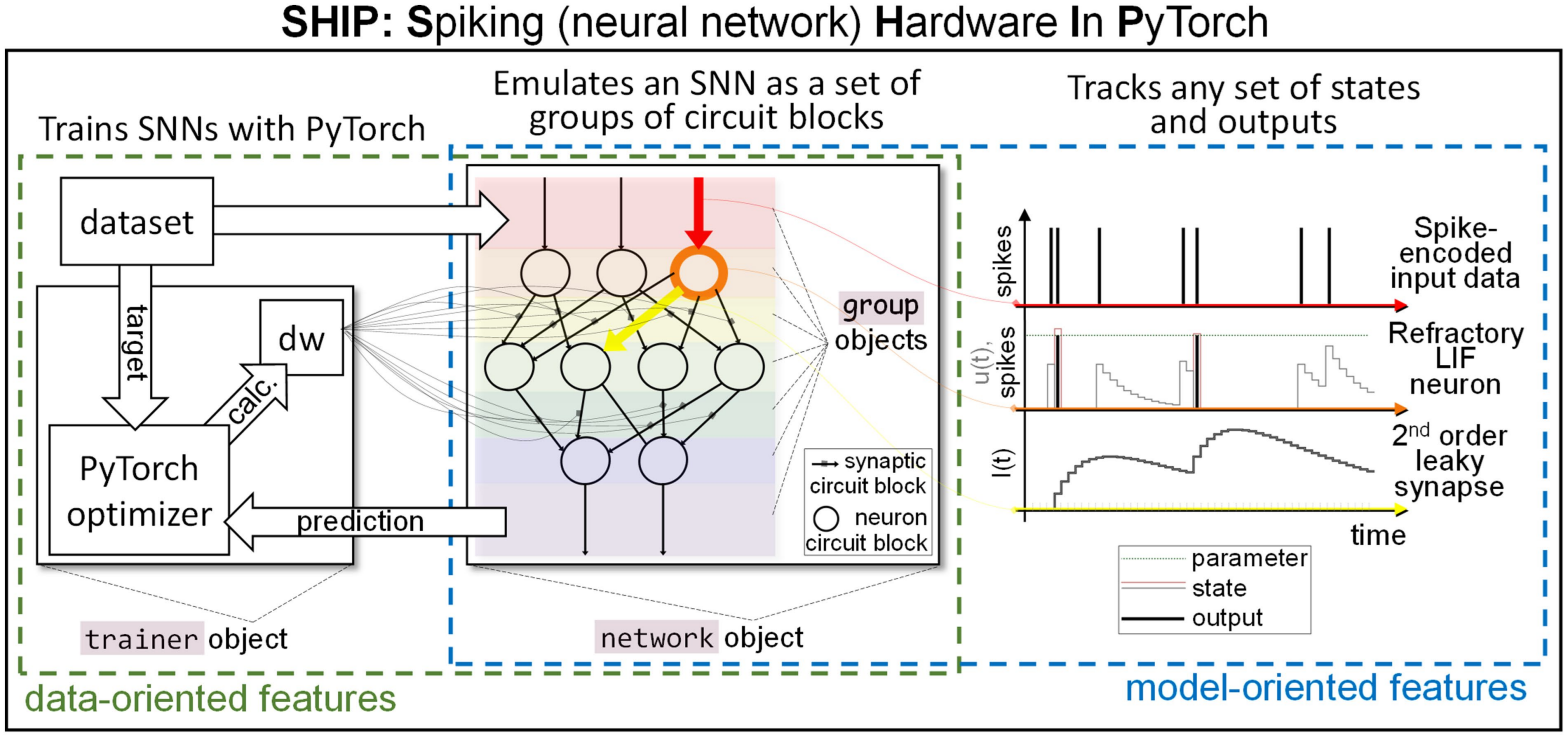
Sketch of our simulation platform, SHIP. A hardware SNN is conceptualized as a set of interoperating groups of circuit blocks (neurons, synapses, etc.); bespoke classes are encoded to support the modeling of groups of circuit blocks, and a network class handles the network building and simulation operations. Any time-resolved state and output can be tracked, facilitating a model-oriented analysis of the behavior of the network (or individual component). A trainer class is also available to support data-oriented simulated experiments, in that it interfaces the simulated SNN with PyTorch-based training algorithms.

We underline that SHIP is instead not meant to simulate generic analog neuromorphic circuits, nor to employ highly accurate material/circuit block models. Its reliance on compact models is specifically suited for simplified simulations and evaluation of novel technologies/concepts at the system level, thus the rationale of SHIP does not overlap with existing physically-realistic emulators. A sketch of the main SHIP features is illustrated in Figure 1. In the central panel, we illustrate how an SNN is conceptualized as a collection of groups of circuit blocks. Each group uses easily-editable class models. Any of the SNN states and outputs, defined within the group models, can be monitored during the simulation task. To exemplify this functionality, on the right panel we plot the time-resolved state/output calculated of one selected component of the first three groups. Namely, we detail the spike-encoded input (red), a refractory leaky-integrate and fire neuron (orange), and a 2nd order leaky synapse (yellow). SHIP also provides data-oriented features, precisely the functionality to train the synaptic weights of the simulated network, leveraging the surrogate gradient technique and conventional machine learning techniques. Here, a dedicated object incorporates the necessary algorithmic steps to apply PyTorch optimization routines onto the simulated SNN (see left panel).

This manuscript is structured into 3 main sections. Section 2 provides the scientific background for this work, intended to guide the reader toward the understanding of the main aspects and challenges of the SNN simulation (by way of compact modeling approach). Section 3 illustrates in depth our developed platform, SHIP, detailing the various algorithmic choices that support the simulation of SNN systems. Section 4 reports on the use of SHIP for a few test cases, to demonstrate the use of this tool with plausible datasets. In Section 4 we will also demonstrate the use of the seamless interface allowing to train the synaptic weights of a simulated SNN through PyTorch.

The source code of SHIP is available at https://github.com/EmanueleGemo/SHIP.

## 2 SNN simulation: key concepts, algorithmic challenges, and available solutions

This section details the SNN simulation challenges and informs about the suitability of a given platform for any intended simulated task. Section 2.1 covers the main algorithmic elements and challenges that emerge from the SNN modeling task. Section 2.2 pictures an intuitive classification for the simulation platform philosophy and interfaces, and lists the numerical platform available to this date. A summary is provided in Section 2.3.

Before delving into such details, we provide a brief description of the physical elements and information processing in (hardware) SNNs, to set some of the definitions used throughout this work.

A minimal SNN can be described as a collection of neuron circuit blocks, interconnected along a set of synaptic circuit blocks. For sake of brevity, we refer to each of the constitutive circuit blocks of a SNN as *component* (which also includes additional functionalities not here listed, e.g., synaptic plasticity). For any component C in a SNN, we identify component *sources* S and *targets* T (wherein the information propagates as S→C→T ). To provide a graphical explanation, the orange component in Figure 1 (center) sees a red source and a yellow target.

The propagation of the information relies on spikes, i.e., temporally-isolated variations of a physical attribute. Neurons collect and integrate spikes, and deliver output spikes according to the internal *state* (i.e., set of physical attributes strictly localized within the component) and spiking functionality. A graphical example of the main traits of a neuron is provided in Figure 1 (right panel, orange element). Synapses carry the spikes across neurons, imprinting amplitude and temporal transformations according to the synaptic functionality and/or internal state. An example of the synapse state and output is also shown in Figure 1 (right panel, yellow element). The input/output operations on SNNs are performed at the *input port* (red shade in Figure 1, center panel) and *output port* (violet shade in Figure 1, center panel).

The computation task of SNNs can be envisioned as a cascade of *events* (spikes, or temporal transformation thereof). These take place downstream of the SNN input port, along both the spatial and temporal coordinates. The output of a SNN collects the events at the output port. The processing power of SNNs arises from (i) the integration and spiking functionalities of the neuron components; (ii) the network topology, i.e., physical arrangement of the components within the network; and (iii), the amplitude and temporal modulation on the transferred spikes, imposed by the synapse components. Training of the synaptic weights in hardware systems is generally implemented by way of local rules, that mimic the functionalities of biological systems (Burr et al., 2017; Brivio et al., 2019; Tavanaei et al., 2019; Wang et al., 2020; Yamazaki et al., 2022). Nevertheless, the transfer of conventional machine learning techniques onto SNNs is an active and ongoing avenue of research (see e.g., Lee et al., 2016; Rueckauer et al., 2017; Tavanaei et al., 2019; Eshraghian et al., 2021, and Yamazaki et al., 2022), though reasonably applicable only in simulated environments.

### 2.1 SNN simulation and critical algorithmic elements

We here picture how the simulation of a hypothetical SNN can be established, underlining the critical factors that affect performance and numerical precision. We focus on the following key aspects: the implementation of the *model* of the components (Section 2.1.1), the *temporal progress* algorithm (Section 2.1.2), and the management of the *data flow* within the network (Section 2.1.3).

#### 2.1.1 Models

We consider the *model* as the algorithmic description of a component (neuron, synapse, etc.). The model of a component ideally tracks the temporal evolution of both *internal states* S and *outputs* O according to a set of mathematical equations and *inputs* I . Inputs, states, and outputs can be any physical attributes or representative properties calculated at any node of interest in the simulated circuit block.

We use two examples to explore in a few more detail how a model can be algorithmically defined. Platforms such as NEURON (Hines et al., 2020), Brian 2 (Stimberg et al., 2019), or DynaSim (Sherfey et al., 2018) use a differential equation system-based model. In these, the user states the variation of certain quantities in differential form. The equation system is then solved by way of consolidated numerical methods (see e.g., Gupta et al., 1985; Byrne and Hindmarsh, 1987; Higham, 2002; Butcher, 2006). The advantages of the use of ODE systems as a way to define a model are immediacy, and the potential access to highly accurate solutions (though one needs to mind the known issues of numerical instability and precision variability of numerical solvers (Higham, 2002)). While numerical ODE solvers find applicable solutions, they are not necessarily conducive to optimized algorithms; therefore, both result accuracy and algorithm performance can vary greatly.

In contrast, other platforms define models as systems of time-discrete equations. Examples are NEST (Gewaltig and Diesmann, 2007), Nengo (Bekolay et al., 2014), and the many reliant on the PyTorch back-end (c.f. Supplementary Table S1). On one side, this strategy allows one to finely tune the algorithm towards enhanced performance or provision of specific features (e.g., pre-calculation of part of the numerical solutions, processing of conditional statements, integration with the platform, etc.). However, analytical solutions often do not exist for arbitrary problems; consequently, approximations may be needed, at the expense of an increased difficulty of the model statement and solution accuracy.

We note that, regardless of the modeling approach, look-up tables can be used in place of dynamic data generation (see e.g., Ros et al., 2006). This method can significantly speed up the equation-solving stage, though at the cost of increased memory encumbrance and granularity of the yielded results (or decreased accuracy, where interpolation is used). We also add that computational strategies, such as vectorization, employ the model of one component to predict the behavior of sets of hierarchically-identical, though independent, components.

#### 2.1.2 Temporal progress

**Figure 2:**
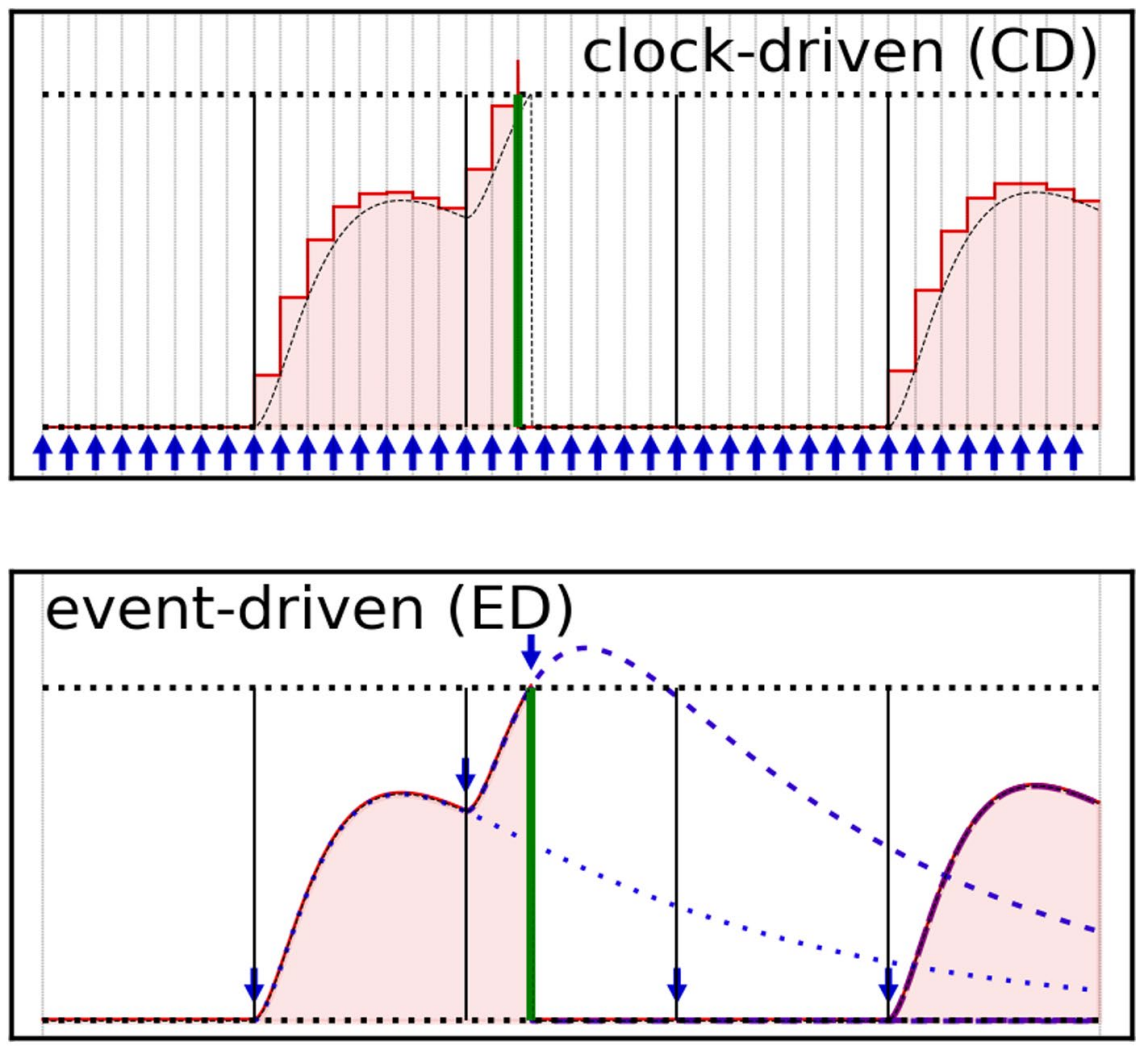
Graphical representation of the clock-driven (CD – top) and event-driven (ED – bottom) time progress approaches, applied to the calculation of a LIF neuron membrane potential. The LIF neuron is subjected to a regular pattern of spikes (vertical lines, solid black) delivered via leaky synapse (not shown). The red line represents the neuron membrane potential that the LIF model would calculate; the area beneath the red line is red-filled, to guide the eye. A green thick vertical bar represents the LIF model output spike. The exact solution is also shown as a dashed black line, for sake of comparison. The horizontal black dotted lines indicate the upper (threshold potential) and lower (rest potential) boundaries for the neuron’s membrane potential. The times at which the simulator updates the numerical solution are indicated with blue arrows. (Top) The CD algorithm mechanically updates the model state and output each time-step (a time-stepped grid is also shown), here generating a set of discrete values (one per time-step). (Bottom) The ED algorithm updates the solution, at each event, potentially reaching the model numerical solution at any point on the (pseudo) continuous time axis. The events at which the ED algorithm updates the solution are both pre-inputted (black lines) or generated (green line). Traces of the partial state updates are also shown (blue dashed/dotted lines).

The temporal progress algorithm drives the advancement through time of the models. Classically, it is possible to distinguish two families of approaches: *clock-driven* (or synchronous), and *event-driven* (asynchronous) algorithms (Tisato and DePaoli, 1995). Hybrid approaches can however be applied under particular conditions. A schematic of the two concepts is presented in Figure 2, in which we track the temporal evolution of the membrane potential of a refractory leaky-integrate and fire (LIF) neuron model (Gautrais and Thorpe, 1998) subjected to a random set of input spikes (black vertical lines), delivered via a 1^st^ order leaky synapse (Eckhorn et al., 1990).

The clock-driven (CD) algorithms quantize the temporal axis in time-steps, and carry out the temporal advancement by iterating through the set of time-steps until the completion of the temporal task, see Figure 2 (top). At each time-step, the CD algorithms call the functions updating the states and outputs of the models, for all the components of a network. We can further classify the CD approaches depending on the availability of mechanisms calculating the time-step size along the simulation. Var*iable* (or *adaptive*) *time-step* CD algorithms bring the advantage of accelerating the temporal progress during time spans in which no event takes place, at the cost of additional computational requirements (though generally not as sizeable as in event-driven algorithms). In contrast, *fixed time-step* CD algorithms forego any computation of the time-step size, and the algorithm can be reduced to the bare minimum by way of a single for-loop.

CD algorithms can generally claim the advantage of simplicity. Additionally, CD methods (especially fixed time-step ones) are more suited for the pre-calculation of part of the solutions for the models, further simplifying the simulation routines. Lastly, CD methods are inherently suitable for parallel computing techniques, as data vectors of equal length (number of time-steps) are used to compute the states and outputs for each model. However, CD algorithms have intrinsic drawbacks. (i) The time-step size is cast to all models, thus leading to oversampling of the “slower” dynamics in order to resolve opportunely the faster ones. (ii) The time quantization imposes a lower boundary on the temporal resolution, which in turn lowers the accuracy and may source computational artifacts. For instance, CD algorithms impose a systematic shift of the spikes at the beginning or at the end of each time-step. In addition, there may be conditions demanding much higher temporal resolution than the one mandated by the dynamics of the components (e.g., lateral inhibition). (iii) Each time-step involves the processing of large vectors, which is resource-burdening if poorly optimized.

We note that multi-clock approaches (seeing locally-defined time quantization for each model) can theoretically mitigate part of the (i–iii) issues. However, these require *ad-hoc* data handling and synchronization between models, introducing some of the drawbacks of the event-driven approach (see following paragraph), and may induce solution granularity. Therefore, multi-clock simulation platforms are uncommon. Due to their strengths and setbacks, CD algorithms remain the way forward for small-to-medium-sized networks in which the dynamic follows predictable patterns, or where the number of time-steps is constrained.

Event-driven (ED) algorithms, in contrast with CD algorithms, drive the temporal advancement forward only when triggered by the variation of any of the inputs/states/output. This methodology potentially curtails the number of iterations through time, and data communication between models (taking particular advantage of sparse vectors, which store only non-zero data and their unit coordinates), leading to the reduction of the average number of operations per point-model carried out by both central unit and memory controller. An initial list of events is constructed from the provided input and only then updated according to the generated states and outputs of the model of each unit. An example is present in Figure 2 (bottom); there, the algorithm updates the state of the model for any sequentially-delivered input, until the model generates a new event (green output spike), and triggers an additional state update. Since ED algorithms do not explicitly rely on the temporal axis quantization, they may find better numerical solutions when compared to CD algorithms reliant on the same model. However, this approach scales poorly with dense spiking patterns, as each event entails a sizeable computational effort, involving localized (hence inefficient) data transfer and time-step-dependent updates of states, outputs, and event list. This drawback stacks with the reduction of the manipulation efficiency of sparse vectors (where employed), as this decreases with their size much faster than in the case of dense vectors.

As ED algorithms are in principle strictly sequential, the use of parallel computing techniques is contingent on the synchronization between modeled components. This is a self-contained computational problem, finding no simple solution but being actively researched (see e.g., Plagge et al., 2018; Pimpini et al., 2022). In summary, event-driven approaches carry out calculations only where the events take place, but each event adds to the model operations the overhead of a set of corollary functions. Therefore, ED algorithms are generally suitable for simulations of SNNs involving sparse spiking.

#### 2.1.3 Data flow

With *data flow*, we refer to the data transfer across the components of a network. As the data exchange between the models is repeated across both unit and temporal coordinates, its management needs careful implementation to avoid wasting clock cycles for unnecessary data handling operations (memory read-write, function calls, etc.). We note that the data flow management also translates algorithmically the causal correlation between the components of a network. Therefore, the management of the data flow is entwined with the temporal progress algorithm (as the causal relation is of course embedded in the temporally-resolved spiking pattern). As such, the data flow management is often dependent on the temporal progress algorithm. Nevertheless, the simulated SNN input–output operations (across its constitutive models) require an *ad-hoc* algorithmic handling, that should support networks of arbitrary topology complexity and sizes.

**Figure 3:**
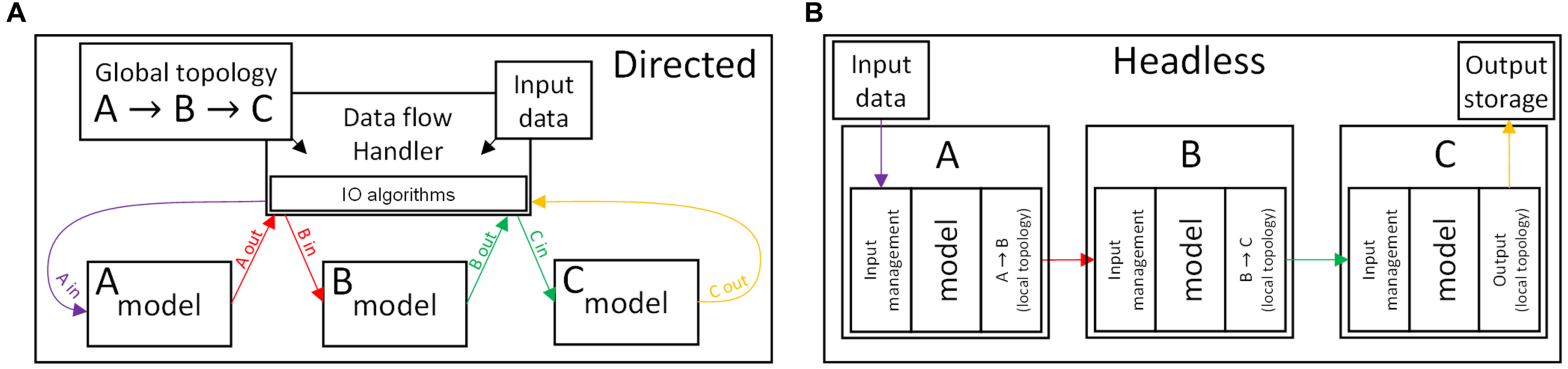
Schematics illustrating two antipodal approaches to the data flow management, for the case of a simple network consisting of the components (A,B,C) and where the data flow follows the sequence *input* → A → B → C → *output*. (Top) Directed strategy, in which the interactions between components are managed by a dedicated handler. (Bottom) Headless strategy, where the components operate independently, and contain both a behavioral model and the IO algorithms.

The main challenge in managing the data exchange between components (or sets thereof) remains the algorithmic optimization. We also note the temporal variation of the network topology as an additional obstacle (Zenke and Gerstner, 2014), which may prevent one from encoding the topology as a static element (contributing to the algorithm complexity and computational burden). There are no classically distinct case scenarios that classify the data flow algorithms. However one may envision how the IO operations can be operated according to a “*directed”* strategy, in contrast to a “*headless”* strategy. The concepts are graphically illustrated in Figure 3, comparing the two strategies for the data flow handling in an example network consisting of three components A, B, and C.

A directed algorithm, see Figure 3A, delegates all the function calls and IO operations to an independently-developed handler, explicitly aware of the topology of the network. A directed algorithm assumes a standardized software-level interface of the models in a network. Consequently, the directed algorithm performs a repeated, predictable set of operations, with added benefit for the simulator platform modularity and simplicity of development. This strategy also facilitates the application of parallel calculation techniques (as it fits seamlessly within the CD techniques, performing the IO operations adjacently to the calculation of the outputs of each model). However, the handler should account for all of the unique cases, exceptions, and conditional operations that the data transfer may entail, and it loses efficiency if burdened by complex software interfaces.

A headless algorithm sees minimal management of the models, which would independently operate in an asynchronous cellular automata-like fashion, see Figure 3B. In this case, the handling of unique cases and exceptions is delegated to the models of the components. Thus, the burden of the optimization of the IO operations falls on the model development. This strategy is therefore more fitting to an ED algorithm, and it can be advantageous as it potentially reduces the overhead contribution due to data-handling function calls. However, this approach is detrimental to the modularity of the simulator (as unique cases must be handled by the user), and may not fit with conventional parallel computation techniques.

### 2.2 Available simulation platforms

**Figure 4:**
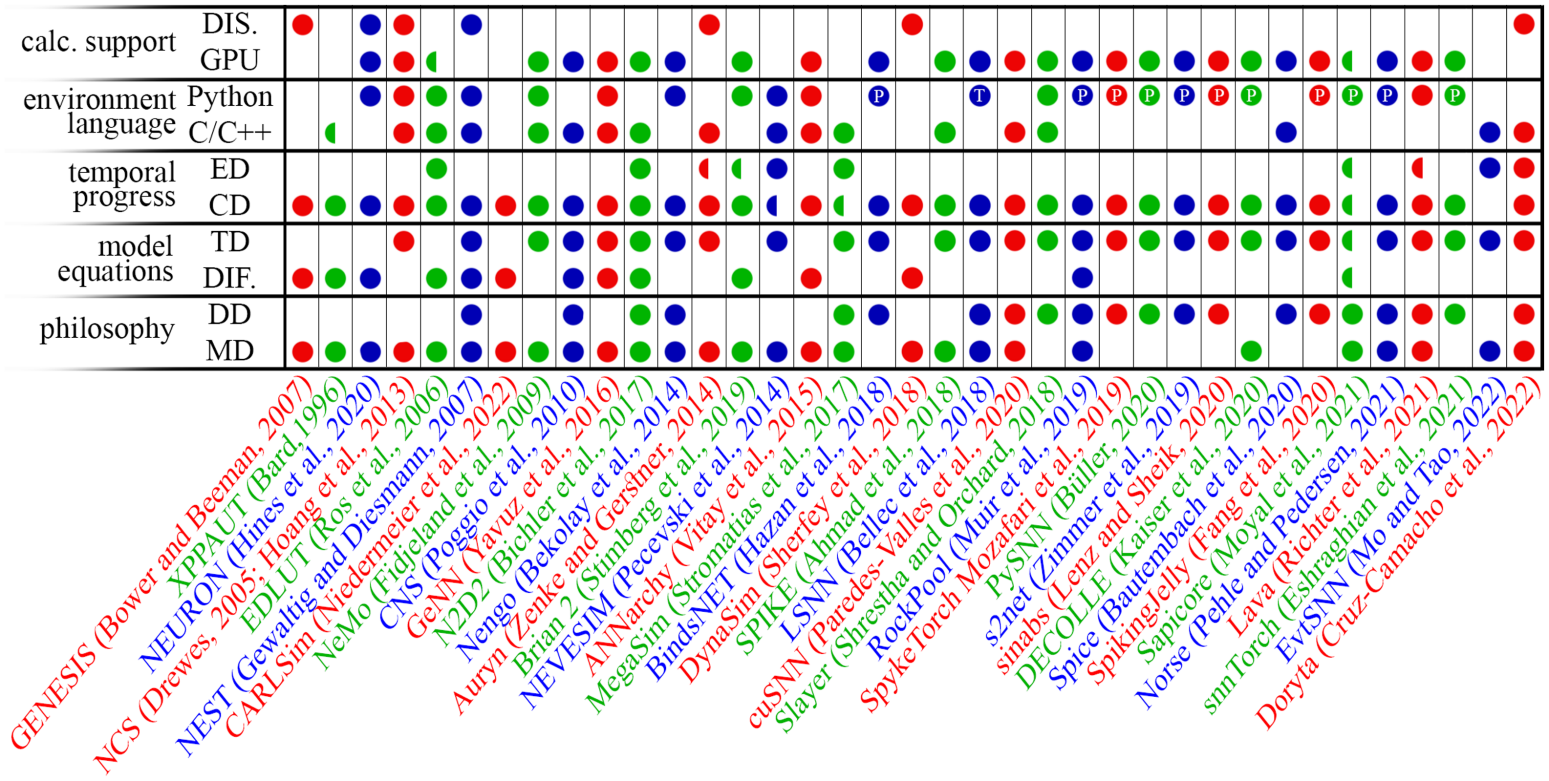
Panoramic of a selection of the available SNN simulation platforms, as of June 2023. Legend: MD – model-driven approach; DD – data-driven approach; TD – time-discrete equation system model; DIF. – differential equation system model; CD – clock-driven temporal handling; ED – event-driven temporal handling; DIS. – distributed computing available. PyTorch and Tensorflow support are also indicated with the symbol P and T respectively, in the environment language field. Half-markers indicate partial support (see Supplementary Table S1 for further information). The list of platform appears in order, from the earliest release to the most recent, and it encompasses the following: GENESIS (Bower and Beeman, 2007), XPPAUT (Bard, 1996), NEURON (Hines et al., 2020), NCS (Drewes, 2005; Hoang et al., 2013), EDLUT (Ros et al., 2006), NEST (Gewaltig and Diesmann, 2007), CARLSim (Niedermeier et al., 2022), NeMo (Fidjeland et al., 2009), CNS (Poggio et al., 2010), GeNN (Yavuz et al., 2016), N2D2 (Bichler et al., 2017), Nengo (Bekolay et al., 2014), Auryn (Zenke and Gerstner, 2014), Brian 2 (Stimberg et al., 2019), NEVESIM (Pecevski et al., 2014), ANNarchy (Vitay et al., 2015), MegaSim (Stromatias et al., 2017), BindsNET (Hazan et al., 2018), DynaSim (Sherfey et al., 2018), SPIKE (Ahmad et al., 2018), LSNN (Bellec et al., 2018), cuSNN (Paredes-Valles et al., 2020), Slayer (Shrestha and Orchard, 2018), RockPool (Muir et al., 2019), SpykeTorch (Mozafari et al., 2019), PySNN (Büller, 2020), s2net (Zimmer et al., 2019), sinabs (Lenz and Sheik, 2020), DECOLLE (Kaiser et al., 2020), Spice (Bautembach et al., 2020), Spiking Jelly (Fang et al., 2020), Sapicore (Moyal et al., 2021), Norse (Pehle and Pedersen, 2021), Lava (Richter et al., 2021), snnTorch (Eshraghian et al., 2021), EvtSNN (Mo and Tao, 2022), and Doryta (Cruz-Camacho et al., 2022).

The elements listed above (and the algorithmic implementation thereof) have a key role in the deployment of the simulation strategy into a cohesive simulation platform. Each algorithmic strategy harbors both advantages and drawbacks concerning flexibility, scalability, abundance of features, performance, and simplicity of use. A highly-efficient and broadly-applicable simulation strategy is not attainable (Prieto et al., 2016; Kulkarni et al., 2021); thus, aimed compromises must always be put in place to balance the intended benefits and the unavoidable costs. As a consequence of the number of possible strategies, the scientific community finds a wide range of simulation platforms that can be readily used for or adapted to the simulation of hardware-based SNNs. However, the choice of a simulation platform may only come after the understanding of the key aspects of each. An early survey of tools applicable to the simulation of SNNs is found in Brette et al. (2007), which directly compares the use and performance of 8 platforms. More recently, Prieto et al. (2016) compiled a more updated and extended list of tools, although not proposing any performance-wise comparison; this is understandably due to (i) the sheer number of takes on the SNN simulation problem, 23 in Prieto et al. (2016); and (ii) the marginal overlap between the target audiences that the proposed platforms address. More recent comparisons between a reduced number of platforms are nonetheless available in literature (see e.g., Tikidji-Hamburyan et al., 2017; Kulkarni et al., 2021). In Figure 4 we propose an up-to-date, though non-exhaustive, panoramic of the proposed numerical tools suitable for SNN simulations. We limit our choices to the numerical tools that are open-source, non-deprecated, offering a maintained or stable version. Supplementary Table S1 (Section 1) provides a detail-enriched version of the data illustrated in Figure 4, which facilitates the in-depth comparison of the technical solutions of each platform to the readers seeking a deeper understanding of the landscape of the numerical tools for SNN simulations. The list of platform appears in order, from the earliest release to the most recent, and it encompasses the following: GENESIS (Bower and Beeman, 2007), XPPAUT (Bard, 1996), NEURON (Hines et al., 2020), NCS (Drewes, 2005; Hoang et al., 2013), EDLUT (Ros et al., 2006), NEST (Gewaltig and Diesmann, 2007), CARLSim (Niedermeier et al., 2022), NeMo (Fidjeland et al., 2009), CNS (Poggio et al., 2010), GeNN (Yavuz et al., 2016), N2D2 (Bichler et al., 2017), Nengo (Bekolay et al., 2014), Auryn (Zenke and Gerstner, 2014), Brian 2 (Stimberg et al., 2019), NEVESIM (Pecevski et al., 2014), ANNarchy (Vitay et al., 2015), MegaSim (Stromatias et al., 2017), BindsNET (Hazan et al., 2018), DynaSim (Sherfey et al., 2018), SPIKE (Ahmad et al., 2018), LSNN (Bellec et al., 2018), cuSNN (Paredes-Valles et al., 2020), Slayer (Shrestha and Orchard, 2018), RockPool (Muir et al., 2019), SpykeTorch (Mozafari et al., 2019), PySNN (Büller, 2020), s2net (Zimmer et al., 2019), sinabs (Lenz and Sheik, 2020), DECOLLE (Kaiser et al., 2020), Spice (Bautembach et al., 2020), Spiking Jelly (Fang et al., 2020), Sapicore (Moyal et al., 2021), Norse (Pehle and Pedersen, 2021), Lava (Richter et al., 2021), snnTorch (Eshraghian et al., 2021), EvtSNN (Mo and Tao, 2022), and Doryta (Cruz-Camacho et al., 2022).

To rapidly capture the philosophy of each of the proposed simulation platforms, we attempted to classify these as ones following *model-driven* or *data-driven* paradigms (though we note that this can be a reductive interpretation; we refer to the provided references for a more comprehensive description of the characteristics of each tool).

The *model-driven* paradigms are intended to gather knowledge on how the simulated networks subtly operate. The interface of a model-driven simulation platform is often supported by a naturally-readable code, and they allow one to easily monitor the temporal evolution of most of the algorithm variables, yet likely sacrificing computational efficiency or facilitated access to off-line training routines. Brian 2 is a representative example, natively oriented to neuro-science experimenters.

In contrast, *data-driven* paradigms seek to obtain the inference and training results with the least amount of computational requirements. Data-driven platforms generally rely on high-level language to rapidly define most of the network and algorithmic features, delegating the eventual access to finer features to a lower-level language. The interface is often not immediately comprehensible to the non-trained user and may lack in flexibility necessary to define *ad-hoc* models. However, the efficiency of data-driven paradigms suits computer-science-oriented investigations. Examples can be found in the more recent proposals that integrate SNN handling functionalities into the PyTorch environment (Mozafari et al., 2019; Zimmer et al., 2019; Büller, 2020; Fang et al., 2020; Lenz and Sheik, 2020; Eshraghian et al., 2021; Pehle and Pedersen, 2021).

Despite the rigid distinction between model-driven and data-driven paradigms, it is common to find software solutions borrowing some characteristics from the two complementary philosophies. Nevertheless, the co-presence of elements of the two philosophies is often limited to a few characteristics, thus not constituting a key aspect of the overall platform. Some noteworthy examples manage to offer a much wider range of features taken from both worlds, see e.g., Lava (Richter et al., 2021) and RockPool (Muir et al., 2019). We find however that, due to the richness of features of these two platforms, dedicated training is due before reaching the proficiency needed to prototype and test new technologies in a simulated network.

### 2.3 Summary

We have underlined the important technical elements that underpin any SNN simulation platform.

Starting from the assumption that the modeling follows a compartmentalized approach, with each model applicable to a component (or a set thereof), we described in detail the main algorithmic elements that drive the functioning of any SNN simulator: the model framework, the temporal progress and the management of the data flow. The model framework may accept ODE systems or sets of time-discrete equations. The temporal progress takes place either via a clock-driven or an event-driven algorithm. The data flow follows a directed or a headless approach. Each of these algorithmic strategies has unique advantages and disadvantages, which determine the platform functionality, features, ease of use, and computational efficiency.

We listed the simulation platforms available to this date, focusing on the open-source ones offering a stable or maintained version. To facilitate the understanding of the features of each, we attempted to classify these based on the philosophy (reflected in features and user interface). Numerical tools can have *model-driven* features, that help in analyzing the behavior of the simulated network. In contrast, *data-driven* features bring the focus to the network output, and where the network itself becomes instrumental to the data processing.

## 3 Method

A simple SNN simulation environment, merging data-driven efficiency and training functionalities with model-driven features can be instrumental for the audience dedicated to hardware SNN prototyping and analysis. It is for this reason that we developed SHIP, which provides the user with the following functionalities: (i) an uncomplicated and easy-to-learn interface; (ii) rapidity of calculation; (iii) facilitated access to a wide range of time-dependent parameters and results; (iv) facilitated development and deployment of user-defined models; (v) accessibility to methods enabling both off-line and on-line training; (vi) suitability to perform parameter-dependent simulations.

This section illustrates the algorithmic strategies that support the functioning of SHIP, the consequential assets, and inevitable drawbacks. In Section 3.1 we provide a synthetic description of SHIP using the concepts illustrated in Section 2. In Section 3.2 we describe the modeling strategy, also including two examples of neuron and synapse models as implemented in SHIP. In Section 3.3 we show how SHIP translates the conceptual network architecture into a manageable sequence of groups of components. In Section 3.4 we further detail how SHIP carries out the SNN simulation, as a result of the integration of the data-flow algorithm, the temporal progress algorithm, and the devised modeling strategy. A summary is eventually provided in Section 3.5.

### 3.1 Base concepts

The main algorithm of SHIP has been developed in Python, due to its relatively simple syntax and widespread use. SHIP uses bespoke classes (datatype containing a template of *properties*, i.e., variables, and *methods*, i.e., functions) for both networks and their constitutive components (neurons, synapses, etc.). The classes for the components include the intended models, and provide their software interface. The class for the network essentially supports the user interface and handles the models of the components during the simulation operations. The numerical handling is instead carried out by a PyTorch backend, that enables SHIP to inherit PyTorch advantages and functionalities: (i) access to optimized libraries enabling fast matricial calculation, (ii) the network optimization algorithms and routines carrying out the network machine-learning-based training, and (iii) the availability of GPU-accelerated calculations.

To fast-track the reader onto the understanding of the inner mechanism of SHIP, we describe our platform using the concepts previously discussed in Section 2. SHIP (i) proposes a model-driven interface to a data-driven back-end; (ii) defines each model as a set of time-discrete equations; (iii) regulates the temporal advancement according to a CD algorithm; (iv) drives the data flow according to a directed algorithm, that has been structured to bear minimum computational overhead (i.e., a for-loop) and yet allows SHIP to simulate virtually any network topology.

### 3.2 Model development

**Figure 5:**
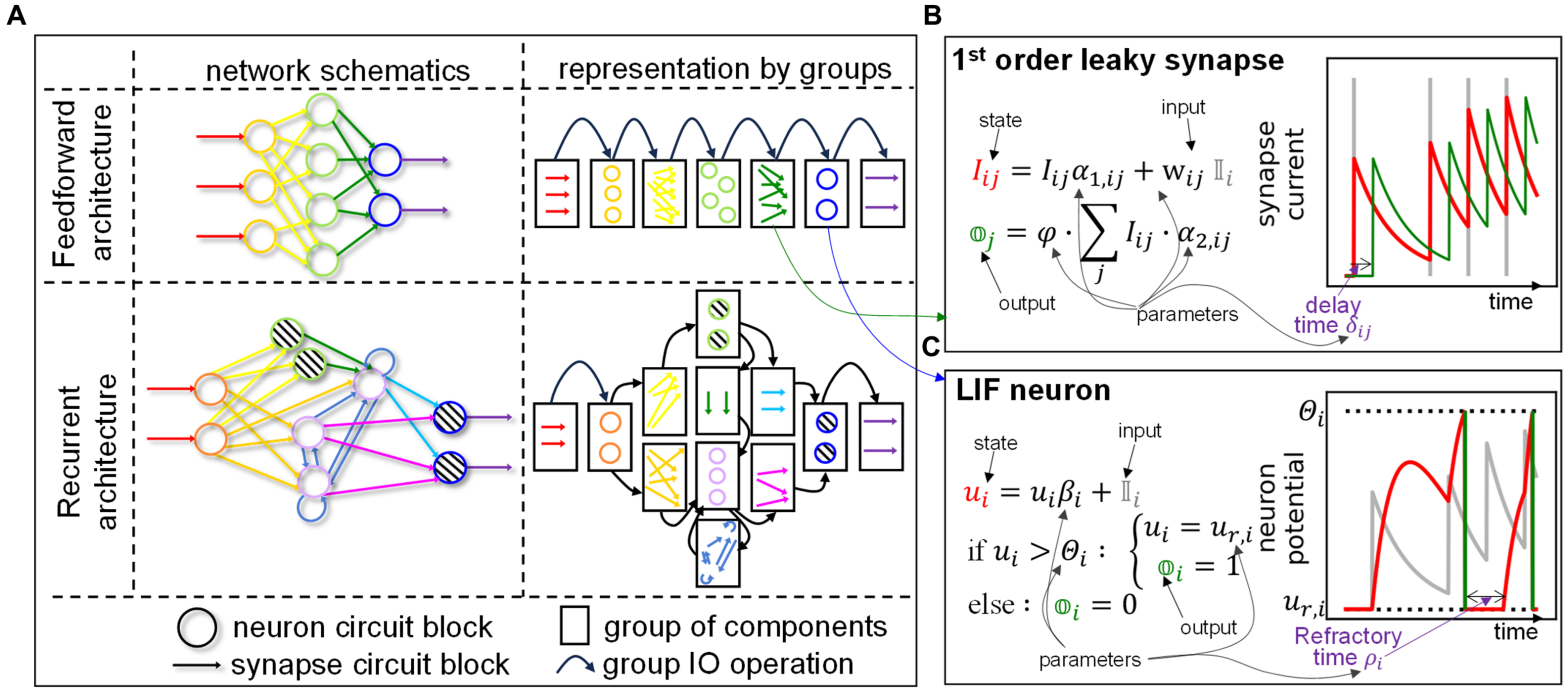
Modeling approach of the SHIP platform. **(A)** Examples of the representation by groups of two different networks: a feedforward one (top) and a recurrent one (bottom). On the left, the network schematics are proposed. Their corresponding representation by groups is shown on the right of the chart. Colors and symbols guide the eye to the identification of the groups and components in both representations. Legend is proposed at the bottom, outside the chart. **(B)** Essential details of the 1^st^ order leaky synapse model, an applicable model for synaptic circuit blocks. The time-discrete equation is shown on the left. A plot on the right exemplifies the state (red) and output (green) variation as a function of an arbitrary input (grey vertical lines). **(C)** Essential details of the LIF neuron model, following the same schematics of the previous example, and whose sample plot is driven by the output of the synaptic model. The $\left(i,j\right)$ subscripts in the equations of (b,c) represent the individual components addressed by each group.

SHIP simulates a network as a collection of interoperating *groups*, i.e., sets of components that (i) share the same model and (ii) are hierarchically equivalent within the network structure (i.e., share sources/targets belonging to the same source/target group). The definition of group is deliberately loose, so as to encompass sets of any components. To further clarify this aspect, a group of neurons matches the definition of *layers* in conventional machine learning. However, groups of synapses (sharing the same source and target neuron sets) or any other arbitrarily chosen component can be defined. The concept is illustrated in the chart of Figure 5A, which shows two examples, a feedforward (top) and a recurrent network (bottom). Two possible network schematics are proposed on the left column. The corresponding representation by groups is shown on the right column of the chart. Each network is represented as a collection of groups (boxes), concatenated as a directed graph (where the arrows symbolize the group IO operations); each group contains a set of components, taken from the network representation on the left.

A model in SHIP regulates the behavior of a group of components, and finds implementation in a dedicated *class*. A group class defines an arbitrary number of properties and methods, but the model is structured through the following template of methods:

- time_dep, which precalculates all the time-step-dependent parts of the solutions;
- set_initial_state (that has a self-explanatory name);
- advance_timestep, that for any time t calculates the set of internal states $S_{t+1}$ and the output $O_{t}$ , as dictated by i) a time-discrete set of equations, ii) the set of internal states $S_{t}$ and iii) the input $I_{t}$ .

We underline that, by design, a model in SHIP represents a set of components (of cardinality equal to or larger than 1). Therefore, its algorithm must rely on vectorization, which is supported through PyTorch.

As mentioned in Section 2.1.1, defining a model as a set of time-discrete equations is less intuitive than the provision of an ODE or PDE system, since it requires the user to carry out the necessary mathematical analysis. However, we deem this compromise acceptable, as in SHIP it is conducive to a much simpler algorithm, with immediate performance benefits. To mitigate this aspect, we provide the easy-to-amend scaffold of methods mentioned above, which (i) clearly explains to the user how to structure new models, and (ii) allows the main algorithm to carry out the simulation task in the most efficient way.

Furthermore, this approach fits particularly well with the intended use of SHIP. Analytically-solvable models (leaky integrate-fire (LIF) neuron, n^th^ order leaky synapse with n≥0 ) can be used to mimic artificial neuron and synapse components with good approximation (see e.g., Bartolozzi and Indiveri, 2007; Chicca et al., 2014; Brivio et al., 2019; Yang et al., 2020; Fang et al., 2022b). We anticipate that we will also explore an example using a non-solved ODE system, the Izhikevic model (Izhikevich, 2003), which is implemented by way of the Forward-Euler approach (see Section 3.2.3).

The behavior of the LIF neuron and the leaky synapse (LS) are summarized in Figures 5B,C respectively. These show the time-discrete equation driving the behavior of the models: state (red) and output (green) variation, as a function of the input (grey). The following subsections further explore these models, as implemented in SHIP (Rotter and Diesmann (1999) contains additional information on the mathematical derivations here proposed). We note that further functionalities can be rapidly added to any SHIP model exploiting class inheritance. Indeed, we deploy the functionalities of delay and refractoriness by using superclasses, that interject the delivery of the output from the parent class and apply optimized post-processing algorithms.

We underline that SHIP does not attribute physical units to variables and parameters (as NEURON or Brian 2 do). This choice delegates the interpretation of the numerical results (and any error-checking of the equations in the model) to the user but provides one with a leaner algorithm execution.

#### 3.2.1 Leaky synapse model

The LS generates a post-synaptic exponentially decaying current transient, upon reception of a pre-synaptic spike. During a time-step dt , a synapse connecting neurons i and j sees the variable current $I_{ij}$ decreasing according to a phenomenological temporal constant $\tau_{\alpha ,ij}$ and increasing according to the synaptic weight $w_{ij}$ upon detection of a spike $\delta_{i}$ from the source neuron i . The state variation for the 1^st^ order model is calculated as follows:


$$I_{ij}\left(t+dt\right)=I_{ij}\left(t\right)\cdot \exp\left(-dt/\tau_{\alpha ,ij}\right)\;+w_{ij}\cdot \delta_{i}\left(t\right)$$

For sake of convenience, the output $O_{j}$ sums up all contributions of the group towards the neuron j (which would more correctly be the role of the dendrites). The output $O_{j}$ also considers the current temporal integration along the time-step duration:


$$O_{j}\left(t\right)=\varphi \cdot \Sigma_{j}I_{ij}\left(t\right)\cdot \tau_{\alpha ,ij}\left(1-\exp\left(-dt/\tau_{\alpha ,ij}\right)\right)$$

with φ being a group-wise, scalar scaling factor. The calculation of state variation and output are indeed collected within the advance_timestep method. The time_dep method instead can pre-calculate the parameters $\alpha_{1,ij}=\exp\left(-dt/\tau_{\alpha ,ij}\;\right)$ and $\alpha_{2,ij}=\tau_{\alpha }\left(1-\alpha_{1,ij}\right)$ , if none of the parameters $\tau_{\alpha ,ij}$ varies along the simulated inference (otherwise, a multi-stepped inference method can be used). Trivial modification can be applied to the model to remove the temporal integration. A graphical example of the state and output of this model can be viewed in the plot of Figure 5B, as a function of an arbitrarily chosen spiking input (gray lines). The example of Figure 5B also shows the effect of the delay, mentioned before, which shifts the model output by a time δ along the temporal axis. It is important to note that the synaptic groups are always considered fully connected. The pruning of synaptic connections can be carried out by setting the corresponding weights to 0, as a zero-valued parameter $w_{ij}$ sets to 0 the effect of any incoming input $\delta_{i}$ (see the equations above).

We add that a 2^nd^ order model can be reasonably implemented separating and solving independently for the two exponential contributions. The equations above would need to be amended as follows:


$$\left\{ \begin{array}{c} I_{ij}^{+}\left(t+dt\right)=I_{ij}^{+}\left(t\right)\cdot \exp\left(-dt/\tau_{\alpha ,ij}^{+}\right)\;+w_{ij}\cdot \delta_{i}\left(t\right)\\ I_{ij}^{-}\left(t+dt\right)=I_{ij}^{-}\left(t\right)\cdot \exp\left(-dt/\tau_{\alpha ,ij}^{-}\right)\;-w_{ij}\cdot \delta_{i}\left(t\right) \end{array}\right.$$

$$O_{j}\left(t\right)=\varphi \cdot \Sigma_{j}\left(\begin{array}{l} I_{ij}^{+}\left(t\right)\cdot \tau_{\alpha ,ij}^{+}\left(1-\exp\left(-dt/\tau_{\alpha ,ij}^{+}\right)\right)\\ +I_{ij}^{-}\left(t\right)\cdot \tau_{\alpha ,ij}^{-}\left(1-\exp\left(-dt/\tau_{\alpha ,ij}^{-}\right)\right) \end{array}\right)$$

where the + / − suffixed variables track the positive/negative contributions to the synaptic current dynamics.

#### 3.2.2 Leaky integrate-fire neuron model

The LIF neuron sees the internal variable action potential u decreasing according to the dynamics imposed by the temporal constant $\tau_{\beta }$ , and increasing according to the pre-neuronal synaptic current (integrated during the time-step dt ), S, as follows:


$$u_{i}\left(t+dt\right)=u_{i}\left(t\right)\cdot \exp\left(-dt/\tau_{\beta ,i}\right)\;+S_{i}\left(t\right)$$

As done with the synapse model, the exponential term can be precalculated by the time_dep method. The model output O is instead determined as follows:


$$O_{i}\left(t\right)=0\;\mathrm{if}\;u_{i}\left(t\right)<\Theta_{i},\mathrm{else}:O_{i}\left(t\right)=1$$

with $\Theta_{i}$ being a threshold potential scalar value. In case of a spiking event, the model also resets the action potential $u_{i}$ to the rest potential $u_{r,i}$ :


$$u_{i}\left(t+dt\right)=u_{r,i}\;\mathrm{if}\;O_{i}\left(t\right)=1$$

A graphical example of the state (red line) and output (green bars) of this model are presented in Figure 5C, as a function of the synaptic current (gray line), previously calculated and shown in the plot in Figure 5C. The example also shows the effect of the refractoriness, which negates the integration along the time ρ after a spike generation. In SHIP, this effect is implemented by way of a constant $\Pi_{i}$ , that is required to multiply the value of the action potential variation. The neuron model also sets Π to 0, after a spike. The refractory superclass adds to the neuron model the algorithmic steps that maintain Π to the value of 0, until the post-spike time ρ elapses.

#### 3.2.3 Izhikevic neuron model

The Izhikevic model (Izhikevich, 2003) is a commonly found non-linear example, which we can use to demonstrate the applicability of SHIP to modeling non-linear components. It also finds hardware implementation examples (see e.g., Fang et al., 2022a). As the ODE solution is not trivial, we base our implementation on the Forward-Euler method. To determine the state of the system, the model calculates the following:


$$v_{i}\left(t+dt\right)=v_{i}\left(t\right)+h\left(140+\left(5+0.04v_{i}\left(t\right)\right)v_{i}\left(t\right)-u_{i}\left(t\right)+S_{i}\left(t\right)\right)$$

$$u_{i}\left(t+dt\right)=\left(1-\mathrm{ha}\right)u_{i}\left(t\right)+habv_{i}\left(t\right)$$

We note that the parameter h is an adimensional parameter driving the numerical stability (in the Forward-Euler approach the above calculations are performed iteratively for k times, until kh≥1 ).

The output of the Izhikevic model is calculated like the one of the LIF model; the post-spike reset instead follows the equations below:


$$\mathrm{if}\;O_{i}\left(t\right)=1:\{\begin{array}{c} v_{i}\left(t+dt\right)=c\\ u_{i}\left(t+dt\right)=u_{i}\left(t\right)+d\; \end{array}$$

The parameters a,b,c,d determine the model dynamic behavior according to the original statement of the author. We note that refractoriness is here embedded in the model dynamics, and can not be explicitly set with a single variable (in contrast to the LIF model).

### 3.3 Group ordering

The network building stage requires the user to define the groups composing a network (more details will be provided in Section 4.1). When adding the synaptic groups, the user is also tasked to state their respective source and target groups; consequently, the user gradually builds the hierarchical relationships among all groups, without the need to provide an explicit structure of the network prior to the simulation task. Borrowing the concept of node and edge from the mathematical notion of *graph*, the algorithm sees the user-defined network as an unsorted list of *nodes* (neuron groups) and *edges* (synapse groups). We anticipate that SHIP would carry out the inference by the sequential calling of the advance_timestep method, for each group. Therefore, it is necessary to transfer the conceptual SNN graph, implicit in the unsorted list of groups, onto an explicit uni-dimensional representation as a sorted sequence of groups, hereby referred to as *stack*.

**Figure 6:**
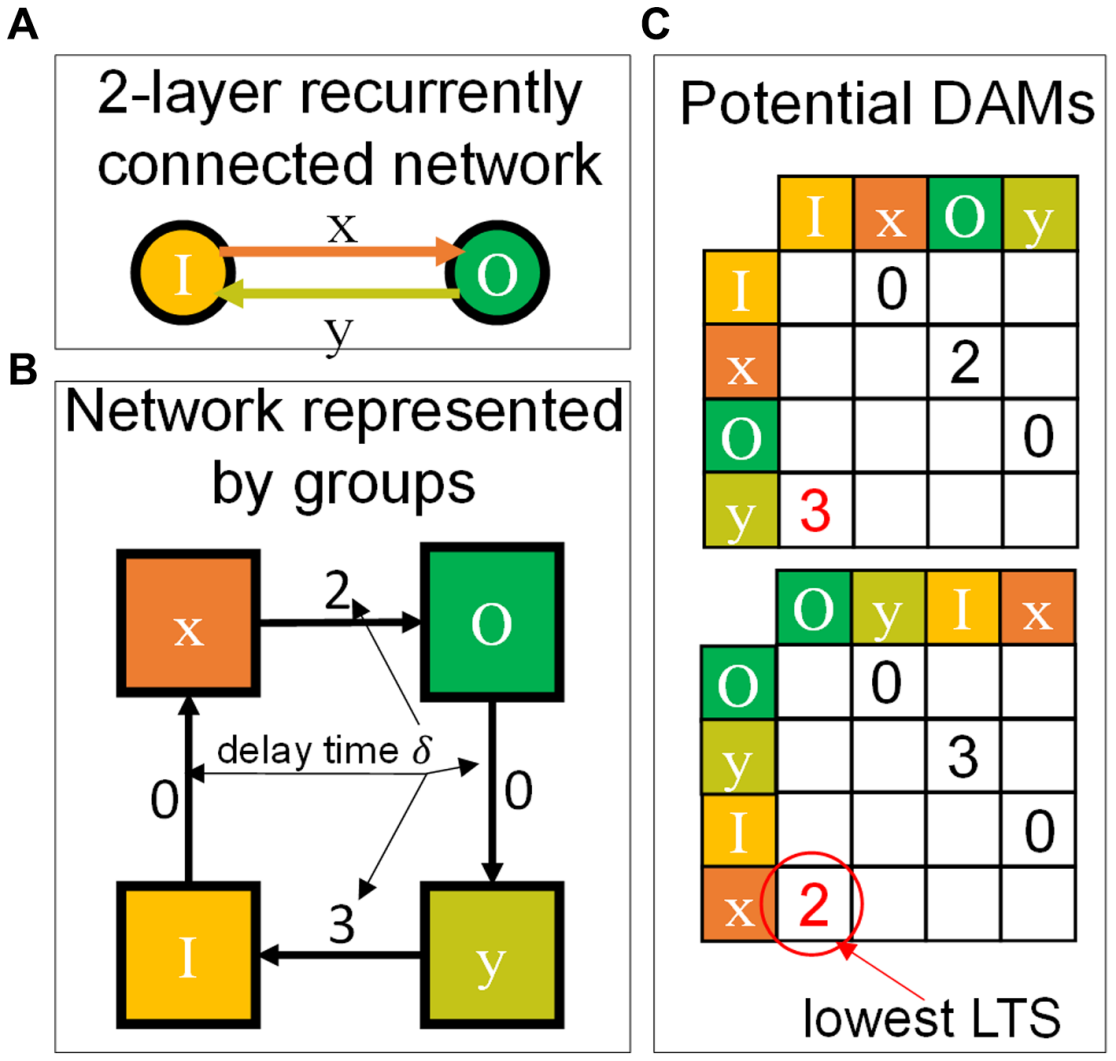
Graphical conceptualization of the process determining the optimal stack sequence. **(A)** Network structure, consisting of an input layer (I) and an output layer (O), connected bidirectionally by the synaptic sets (x,y). **(B)** Representation of the network by groups, reporting arbitrarily chosen delay time values for each connection between groups. **(C)** Possible delay-substituted Directed-Adjacency matrices (DAMs), for the sequences (IxOy) and (OyIx). The lower-triangular sums (LTS) are highlighted in red. The obtained values highlight (OyIx) as the most suitable stack.

Here follows a description of the method used to calculate the stack (carried out once with negligible computational requirement, during the call of the network.init() method). For all possible sorting orders of the groups, SHIP generates a (group-wise) graph representation by way of a directed adjacency matrix (DAM), in which each row (and column) corresponds to a unique group. We note that non-zero values within the lower-triangular section of the DAM break the causal correlation, as they engender an input request that would only follow the respective output provision. Thus, one objective is the minimization of the lower triangular sum (LTS) of the DAM. We add that each connection between groups implies a certain *temporal delay* $\delta_{t}\geq 0$ , which we need to take into account to find the best-fitting group sort order. To do so, the algorithm replaces the non-zero values of the DAM with the corresponding delay values. Any unvaried value is eventually set to a negative infinitesimal value, to correctly process zero-valued delays. The algorithm then calculates the LTS of the generated DAM configurations, eventually determining the earliest physically-realistic one having the lowest LTS value. Its corresponding sequence of groups is the intended stack. Figure 6 illustrates the concept with an example.

Figure 6A shows a 2-layer network consisting of an input layer (I) and an output layer (O). The connection between the two layers is bidirectional (with (x, y) synaptic groups), thus making this example the simplest, non-trivial network to represent as a unidimensional stack. Figure 6B highlights this, by way of the network representation by groups, that form a loop without clear indication of any extremal group of the stack. Figure 6B also reports arbitrarily chosen values for the delay time [a.u.], so to build the potential delay-substituted DAMs. Two significative examples are reported in Figure 6C, for the sequences IxOy (top), and OyIx(bottom). The LTS for the second case reports the lower value, thus indicating that this second stack sequence is more suitable than the counterintuitively logical sequence IxOy. The method described to calculate the stack is redundant for feedforward networks, but we find it useful for recurrent architectures (as shown in the example of Figure 6), as this strategy allows one to use the same models for both feedforward and recurrent architectures with no amendment required. Supplementary material (Section 2) provides more details on the rationale of the objective function of our sorting algorithm.

### 3.4 Network simulation: combining data flow and temporal progress

The temporal progress in SHIP follows a fixed time-step CD algorithm, according to the user-provided time-step size and number of time-steps. This approach allows us to carry out the temporal task by use of a single for-loop iterating through the sequence of time-steps. As mentioned in Section 2.1.2, this approach minimizes overhead and becomes extremely efficient for time-step-limited simulations. A multi-time-step method is also available, which becomes convenient for the user who seeks to change the time-step across the simulation.

The data flow management relies on the definition of the stack. Before the simulation, the network undergoes an initialization process, in which (i) the stack is calculated; (ii) a temporary storage (TS) variable is pre-allocated; (iii) each group of the stack sees the assignment of a set of memory addresses on the TS, for both inputs and outputs. The algorithm overlaps the input address of each target group with the output address of each source group; consequently, the IO operations carried out during the simulation are limited to the query to the TS at the pre-defined memory addresses, reducing the read-write operations and data transfer to the bare essentials (at the cost of an increased memory footprint). We clarify that, unless retrieved and stored by the express choice of the user (via available methods), the data in TS is overwritten in each loop, limiting the generated data.

**Figure 7:**
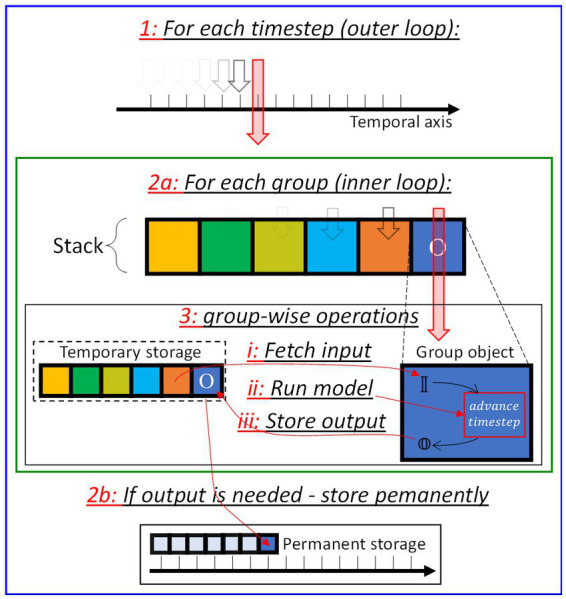
Sketch of the inference algorithm as carried out in SHIP comprising of an outer loop (operation 1, blue box) traversing the temporal axis; an inner loop (operation 2a, green box) traversing the stack, and three main group-wise operations (operation 3i-iii) to be carried out for each group of the stack. After the inner loop, the output is stored if required by the user (operation 2b).

The simulation task is carried out by way of two iterations: (i) an outer for-loop goes through the user-determined set of time-steps; (ii) an inner for-loop traverses the stack sequence, performing the following for each of the groups: (a) retrieval of the input(s) from the TS, (b) call of the advance_timestep method, and (c) storage of the group output onto the TS. The concept is exemplified in Figure 7.

### 3.5 Summary

This section illustrated SHIP, a Python/PyTorch-based platform dedicated to the simulation of hardware SNNs by way of the compact modeling approach, and offering both model-driven and data-driven features. We have shown the key algorithmic strategies chosen to support the simulation task. Additional information on the use of SHIP is available in tutorial format, which is supplied along with the source code.

SHIP simulates a network as an interoperating set of groups. Each group is supported by a model defined as a Python class and is structured to provide an easily-editable set of methods. The model framework uses a set of time-discrete equations. Three widely-adopted models are reported in detail.

The time-resolved simulation of the network is supported by a *clock-driven*, algorithm, in conjugation with a *directed* data flow management that is coded to limit its computational burden. We reported how SHIP linearizes the network graph and we explained how this strategy allows one to simulate any arbitrarily-complex network topology, without requiring to encode bespoke models for each network topology.

## 4 Test cases

To demonstrate how SHIP can be instrumental to SNN design, simulation, and analysis, we here use a few simple test cases. In Section 4.1 we demonstrate how SHIP performs the essential tasks of network building and simulation. In Section 4.2 we show how SHIP can be employed in conjugation with PyTorch to carry out training tasks. In Section 4.3 we describe the use of SHIP applied to the investigation of the architectural-parameter dependency of a trained network accuracy. A brief summary is provided in Section 4.4.

### 4.1 Building a network, performing the simulation, and monitoring the calculated data

As illustrated in Figure 1, SHIP handles the network by use of a dedicated class, also called network. The methods of the network class allow the user to structure the groups of a network, interface a network with the input dataset, and retrieve the deriving results, using a few high-level instructions.

We here list the main methods available with the network class:

- the constructor, that instantiates the network object in memory (i.e., mynetwork = network())
- add, which enables one to add a group to the network object, by provision of (i) the model class, (ii) a unique identifier (id), (iii) and any other argument required to set the group parameters/variables. Using the add method, the user can build the simulated network by sequentially adding neuronal and/or synaptic groups. We note that the addition of any neuron group requires the user to provide its number of components N, whereas the addition of synaptic groups requires the user to state the source group and target group ids. By doing so, the algorithm can dynamically generate both the number of components N for the synaptic group and the overall network topology.
- set_param, which can be used subsequently to the add method to provide or amend any group argument.
- init, which is used to consolidate the provided data and generate all the required internal variables as detailed in Section 3.4, generating the object supporting the intended simulation.
- run, which launches the simulation, and can accept any optional argument as an external input to deliver to the network. We underline that the conventional data structure should be presented in vectorial form, sized according to the [batch size, number of time-steps, number of components]. The eventual simulation output also follows the same convention.
- set_monitor, which uses a wrapper function for the advance_timestep method of the intended group class, that adds a routine to store the simulated results each time-step.
- get_monitored_results, which retrieves the monitored data after simulation.

We remark that the algorithm of SHIP can handle any provided argument for the group objects as a generator function, based on arguments such as the batch size (i.e., number of parallel independent simulations carried out simultaneously) and the number of components. This feature endows the user with full flexibility in dynamically generating any arbitrary distribution of parameters. Further details on this functionality are illustrated in Supplementary material (Section 3).

**Figure 8:**
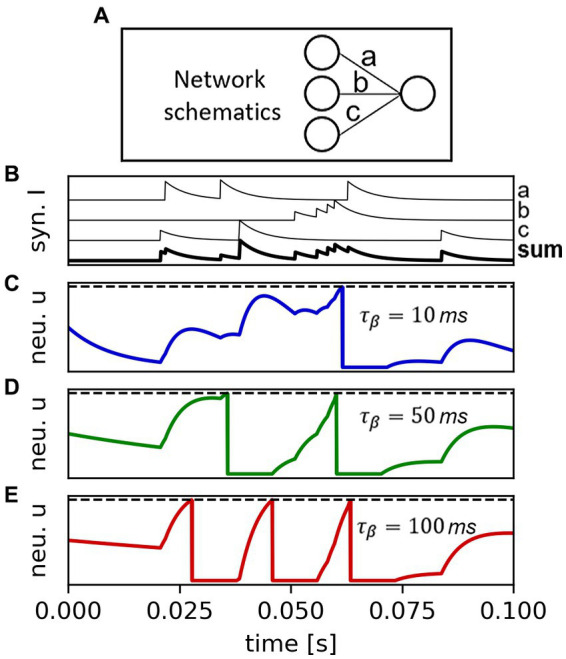
Demonstration of the potential data availability allowed by SHIP, by way of an inference simulation on a simple network (consisting of 3 input neurons, 3 LS synapses, and a LIF output neuron, as shown in **(A)**). **(B)** Time-resolved plot of the single synapse current contributions and of their sum towards the LIF output neuron (data is normalized for sake of convenience). **(C–E)** plot of the membrane potential of the LIF neuron, for the temporal constant values of 10 ms, 50 ms, and 100 ms respectively, here calculated in parallel and obtained simultaneously.

As an example, we show a selection of data that can be effortlessly harnessed during a simulated inference. A simple 2-layer network is instated as shown in Figure 8A (3 input neurons, 1 output neuron), with arbitrarily determined settings. The output neuron uses the LIF class, for which the temporal constant is varied across the parallel simulations from 10 ms to 100 ms with a step of 10 ms. We monitor the synaptic currents, the sum of the currents towards the LIF neuron, and the LIF neuron membrane potential for all the simulated batches. Figure 8B shows the synaptic current values, unvaried for each of the parallel simulations (we report both the single synaptic contributions and their sum). Figures 8C–E show the different response of the LIF neuron as a function of the temporal constant, for three selected cases (10 ms, 50 ms, 100 ms). One can immediately notice the different behavior of the network as a function of the arbitrarily-imposed variation of one architectural parameter, determined using a simple function in the building stage. The code used to generate this data is proposed and further commented in Supplementary material (Section 3). This example, whilst being simplistic, pictures the potential of the application of SHIP for a wider range of analysis, dependent on explicit user-determined conditions.

### 4.2 SNN training via PyTorch-enabled routines

**Figure 9:**
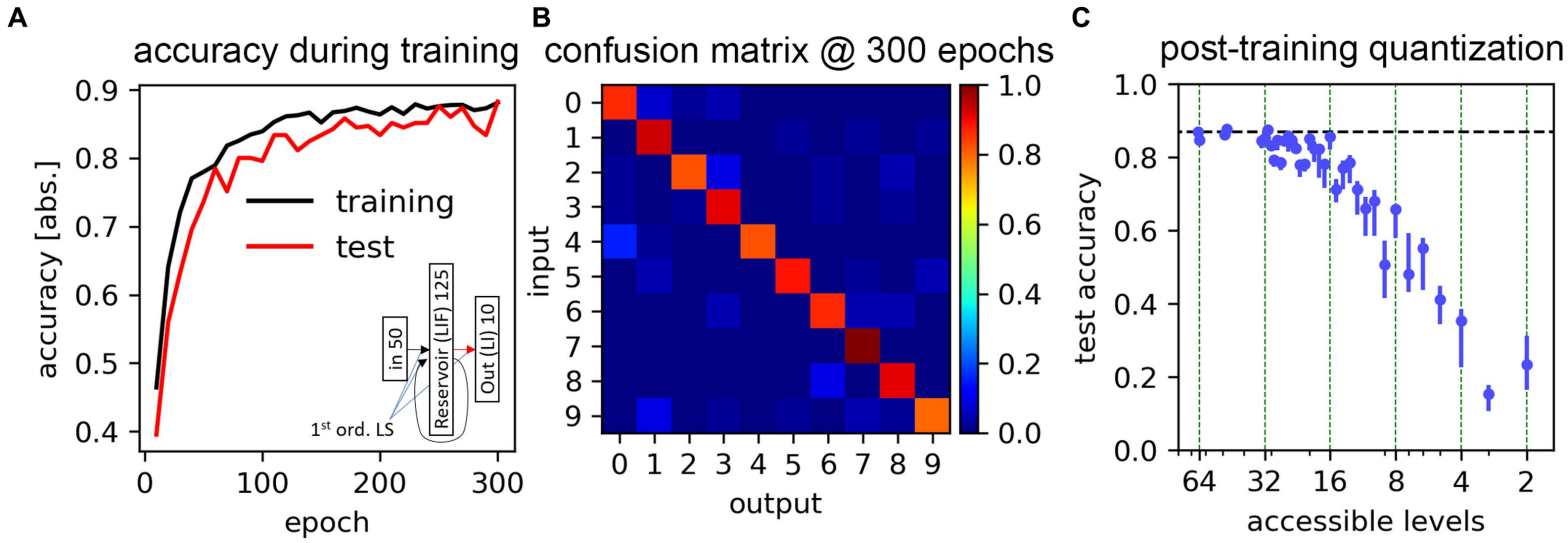
Training results for the classification task of the Free Spoken Digit Dataset. **(A)** Classification accuracy obtained during a training task, with both training and test datasets. The inset shows the adopted network schematics. **(B)** Confusion matrix for the test dataset, calculated at the end of the training process. **(C)** Accuracy results calculated after applying post-training weight quantization, determined using plausible level boundaries and stochastic write error (taken from Pérez et al., 2021), as a function of the hypothetical number of accessible levels. Data averaged on 5 trials. Markers indicate the results average; bars show the min-max range.

We here demonstrate the suitability of SHIP to carry out DNN-based training on SNNs, illustrating the data encoding process, the definition of the SNN architecture, the training technique, and the obtained results. The results reported in this section are also illustrated in Figure 9.

#### 4.2.1 Liquid state machine

We initially demonstrate the feasibility of the training process by use of a *Liquid State Machine* (LSM) (Maass et al., 2002), which is a network relying on a recurrently connected layer (*reservoir*) to unpack the temporally-encoded input onto a unit coordinate (the neurons of the reservoir). The network consists of three layers (input – reservoir – output), with the training being conveniently carried out only on the reservoir-to-output set of synaptic weights.

The dataset of choice is the Free Spoken Digit Dataset (Jackson et al., 2018), containing analog recordings of 3,000 digit utterances from 10 English speakers. The analog-to-spike conversion follows consolidated numerical methods finding plausible physical implementation: Lyon’s cochlear model (Lyon, 1982), conjugated with a bespoke, thresholded (20% of the maximum dynamic range), linear intensity-to-rate conversion algorithm (from 0 to 500 Hz). 85% of the dataset is randomly chosen as a training dataset, whereas the remaining 15% is used as a test dataset.

The use of the LSM method is a widely-accepted SNN architecture for speech-recognition tasks (Verstraeten et al., 2005; Zhang et al., 2015; Gorad et al., 2019; Deckers et al., 2022). The details of the network architecture follow the ones illustrated in Gorad et al. (2019). The resulting network is composed of 6 groups: 50-sized input neurons, 125-sized reservoir LIF neurons, and 10-sized LI neurons; a 6,250-sized LS input-to-reservoir group (200 non-zero valued), a 15,625-sized LS reservoir-to-reservoir group (~2,300 non-zero valued), and a 1,250-sized LS reservoir-to-output group. A schematic of the network is found in the inset of Figure 9A. Following the specifics provided in the reference material, we set the temporal constant of each neuron to 64 ms, and the ones of the synapses to 8 ms.

For this task, the determination of the excitatory and inhibitory neurons of the reservoir, and the deriving synaptic weights of both input-to-reservoir and reservoir-to-reservoir synaptic groups are predetermined by way of bespoke routines external to SHIP (which follows the original author’s indications), to then be passed as static arguments to the add method (c.f. Section 4.1).

We reiterate that, whilst being purely numeric, the modeled SNN is intended to support the simulation of practical devices. As previously stated in Section 3.2, we here use LIF and LS models, for which plenty of practical circuit designs are available in the literature. The scientific literature also offers solutions for both inhibitory and excitatory mechanisms, and for the necessary control of the neuronal and synaptic temporal dynamics. As an example, the authors of (Chicca et al., 2014) combine LIF and LS dynamics enabling both excitatory and inhibitory processes, using conventional CMOS technology.

SHIP provides a useful interface to the PyTorch routines. An *ad-hoc* trainer class has been deployed with the specific scope to enable off-line training. It contains the essential methods to (i) “pair” a trainer with an available network object (defining the trainable parameters, i.e., synaptic weights); (ii) retrieve and postprocess the simulated output; (iii) submit the inference result to the available PyTorch optimization models. In little more detail, the trainer object substitutes the backward method of the neuron groups with a differentiable surrogate gradient, which allows conventional backpropagation techniques to be carried out (here, we employ the method proposed in Neftci et al. (2019)). The trainer class has been developed bearing in mind modularity, simplicity of use, and an easy-to-understand user interface. The available trainer, by default, performs credit assignment based on the peak of the membrane potential of the output neurons (Hu and Liao, 2022), hence our use of LI neurons in place of LIF neurons. This, in turn, is used along with a log-likelihood loss function (Pfister et al., 2006) as an argument for the PyTorch optimizer ADAM (Kingma and Ba, 2015).

The inference is carried out using a time-step size of 1 ms, more than sufficient to track the dynamics of the neurons (with a temporal constant of $\tau_{\beta }$ = 64 ms). Training is carried out for 300 epochs (the totality of the training dataset is presented each epoch), with a batch size of 16. The task has been carried out 10 times, upfront of a change of the randomization seed. The best-case scenario is here reported, with the training and test accuracy presented in Figure 9A, which demonstrates the effect of the training process. The confusion matrix, obtained after the last epoch with the test dataset, is shown in Figure 9B.

The achieved end-of-training accuracy peaks at 88.2%, with an average value of 86.9%. This result approaches the ~91% limit posited for the use of 1^st^ order LS models (Saraswat et al., 2021). We deem this result to confirm the suitability of the proposed platform for seeking SNN training based on conventional machine learning techniques.

To further explore the feasibility of the deployment of such simulated architecture in hardware, we simulate the post-training quantization of the synaptic weights, according to experimentally-determined conductivity distributions for memristor-based synaptic circuit blocks. The cumulative distribution functions provided by Pérez et al. (2021) point to a regular segmentation of the accessible weight levels, and reveals an approximatively normal statistical deviation of each level. We mimic the features of the experimental data, quantizing the trained weights according to a set of equidistant levels (chosen as a symmetric, signed distribution), in which the overlap of the stochastic distribution between each neighboring level is set to 0.3% (i.e., level distributions cross at 3 σ ). Data is reported in Figure 9C. As expected by other literature results (Nagel et al., 2021), our obtained results point to a rapid and progressive reduction of the network accuracy as the number of accessible weight levels decreases. The accuracy approaches the full-precision weight results up to 4 bits (16 levels); at 3 bits, the accuracy already drops at roughly 60%, and at more drastic quantization levels (1 or 2 bits) the system fares barely above chance. Nevertheless, we underline that quantization-aware training techniques (Li et al., 2017) would certainly improve such results, eventually leading to a better performance characterization, and potentially co-design, of the hypothetical SNN.

#### 4.2.2 Multi-layer, recurrent network

Here we carry out the training of a less trivial network, to further demonstrate the applicability of SHIP for a wider range of SNN training tasks, with a network employing non-linear models in place of the previously-illustrated ones. We train an SNN for the classification of a Braille character reading from a linear arrangement of pressure sensors, traversing approximatively orthogonally a single braille character.

We use an image dataset (Braille Character Dataset, 2020), containing 60 grayscale, 28 × 28-sized pictures of each of the 27 Braille characters. Each picture is spline-interpolated to upscale the temporal resolution from 28 to 300 time-steps, and then converted to a time-dependent spike map via delta-encoding (Corradi et al., 2019; Muller-Cleve et al., 2022) using a delta value of 0.01. The converted dataset consists of the analog reading of the differential response of the linearly-arranged sensors. The number of classes is limited to the initial 10 for the sake of task simplification. 500 samples are randomly selected for the training dataset; the remaining 100 are used as the test dataset.

**Figure 10:**
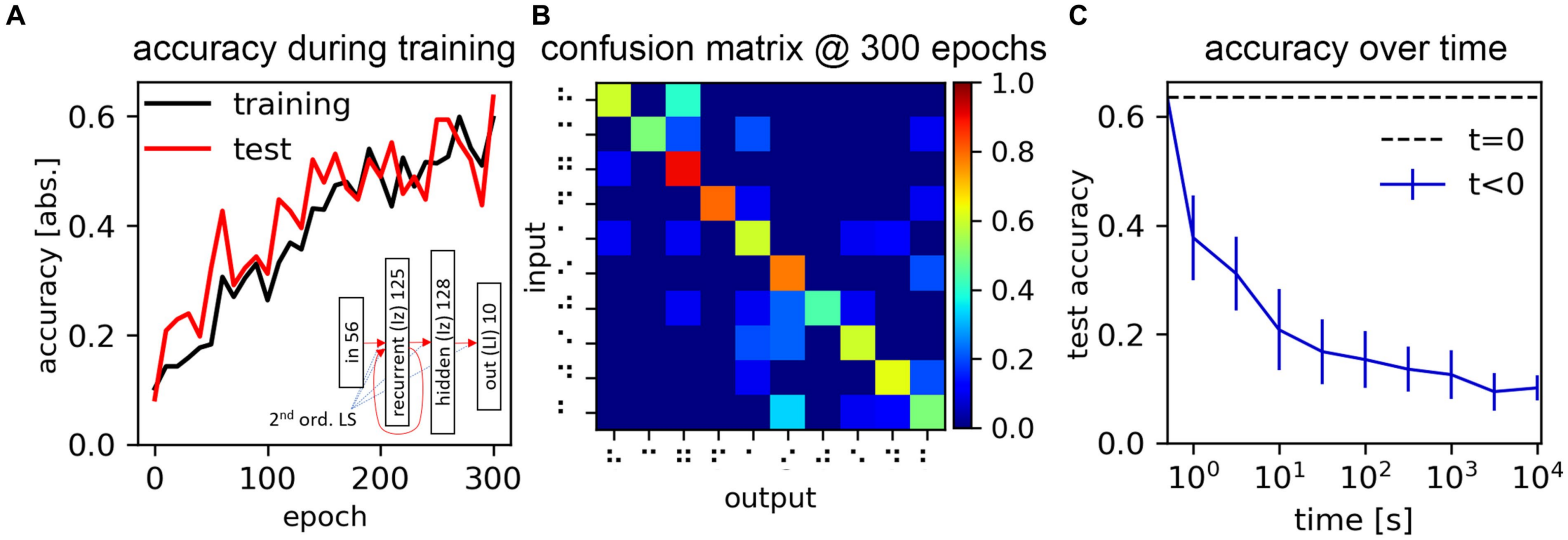
Process and results of the Braille dataset classification task. **(A)** Training and test dataset accuracy, measured during the training process. The inset shows the adopted network schematics. **(B)** Confusion matrix obtained at the end of the training process. **(C)** Network accuracy, as a function of the time elapsed from the parameter storage, due to the drift mechanism of the assumed memristive synaptic weight physical support (model derived from Esmanhotto et al., 2022).

The network of choice consists of an input layer (I) of 56 input neurons; a recurrent layer (R) of 125 Izhikevic neurons; a hidden layer (H) of 128 Izhikevic neurons; and an output layer (O) of 10 LI neurons. A network schematic is proposed in the inset in Figure 10A. The synaptic connections use 2^nd^ order leaky synapses. Like in Section 4.2.1, the training is based on the credit assignment proportional to the internal state value of the output neurons. The synaptic weight initialization of the I-to-R, and R-to-R, follow the procedure illustrated for the LSM in Section 4.2.1; the remaining synaptic groups are initialized with a uniform weight distribution from −0.4 to 0.6. The Izhikevic model sees the parameters a,b,c,d, and Θ as 0.02, 0.2, −65, 8, and 30 mV, respectively. The 2^nd^ order leaky synapses instead follow dynamics imposed by a positive time constant $\tau_{\alpha }^{+}$ = 15 ms, and a negative time constant $\tau_{\alpha }^{-}$ = 5 ms. The hyperparameter optimization addressed the scaling factor of the synaptic groups, which are eventually set to the values of 1,000, 1,500, 800, and 100 for the I-to-R, R-to-R, R-to-H, and H-to-O groups, respectively.

For this task, training affects all synaptic groups, following the routine explained in Section 4.2.1. The batch size is set to 16, and the number of epochs to 300. The training and test accuracies during the training process are reported in Figure 10A. The confusion matrix obtained at the end of the training process is reported in Figure 10B. We measure a peak test accuracy of 63.5%, which may certainly be improved, but demonstrates the effectiveness of our platform in supporting the DNN-based training of an arbitrary SNN network. The confusion matrix also shows a clear diagonalization of the non-zero values.

We here illustrate how the network accuracy may change, assuming the drift of a memristive memory device serving as the physical support of the synaptic weight. We use the circuit design and experimental data presented in Esmanhotto et al. (2022) to derive a drift compact model (more details on the drift model are found in Supplementary material (Section 4)). We updated the synaptic model introducing this mechanism, and we simulated the inference on the test dataset as a function varying the time elapsed from an assumed network parameter storage carried out in a write-verify schema (thus, not reliant on any weight quantization). We measured the network accuracy as a function of the elapsed time. The average and standard deviation (calculated out of 20 random iterations of this simulation) are reported in Figure 10C. Our results show that the network sees a rapid decay of its accuracy, evidencing that a potential hardware implementation of this simulated system would need to address this factor to serve any practical use.

### 4.3 Post-training network analysis

We here use a different case scenario to demonstrate how SHIP facilitates the analysis of the simulated SNN, as a function of the SNN architectural parameters and training results. This task combines SNN training and post-training monitoring of its inner states during inference. In particular, we here train an LSM based on the one detailed in Section 4.2.1, for classification purposes as a function of (i) the temporal constant of the reservoir neurons $\tau_{\beta }$ , and (ii) the reservoir-to-reservoir synaptic scaling factor φ (c.f. Section 3.2.1). The choice of the two parameters was determined via explorative simulations, as these have the largest impact on the classification accuracy.

We use the MIT-BIH Arrhythmia Database (Moody and Mark, 1990), which presents a wide dynamic variation that is useful for our investigation of dependency on $\tau_{\beta }$ of the SNN performance. The dataset contains digitized analog traces, here split in single heart-beats, each composed by 4 independent channels. We convert the analog signals to spiking signals using the delta-encoding technique proposed in hardware in Corradi et al. (2019), however here using a 3-bit scheme in place of a single-bit scheme to further reduce the simulation computational requirement. Since (i) the data conversion determines 12-channel inputs, and (ii) the dataset contains 18 classes, we modify the previous SNN to adapt it to the expected inputs and outputs, imposing a 12-input neuron layer and an 18-LS neuron output layer. The $\tau_{\beta }$ value for the neurons of the reservoir group is initially set to 1 s. Training follows the technique detailed in Section 4.2.

**Figure 11:**
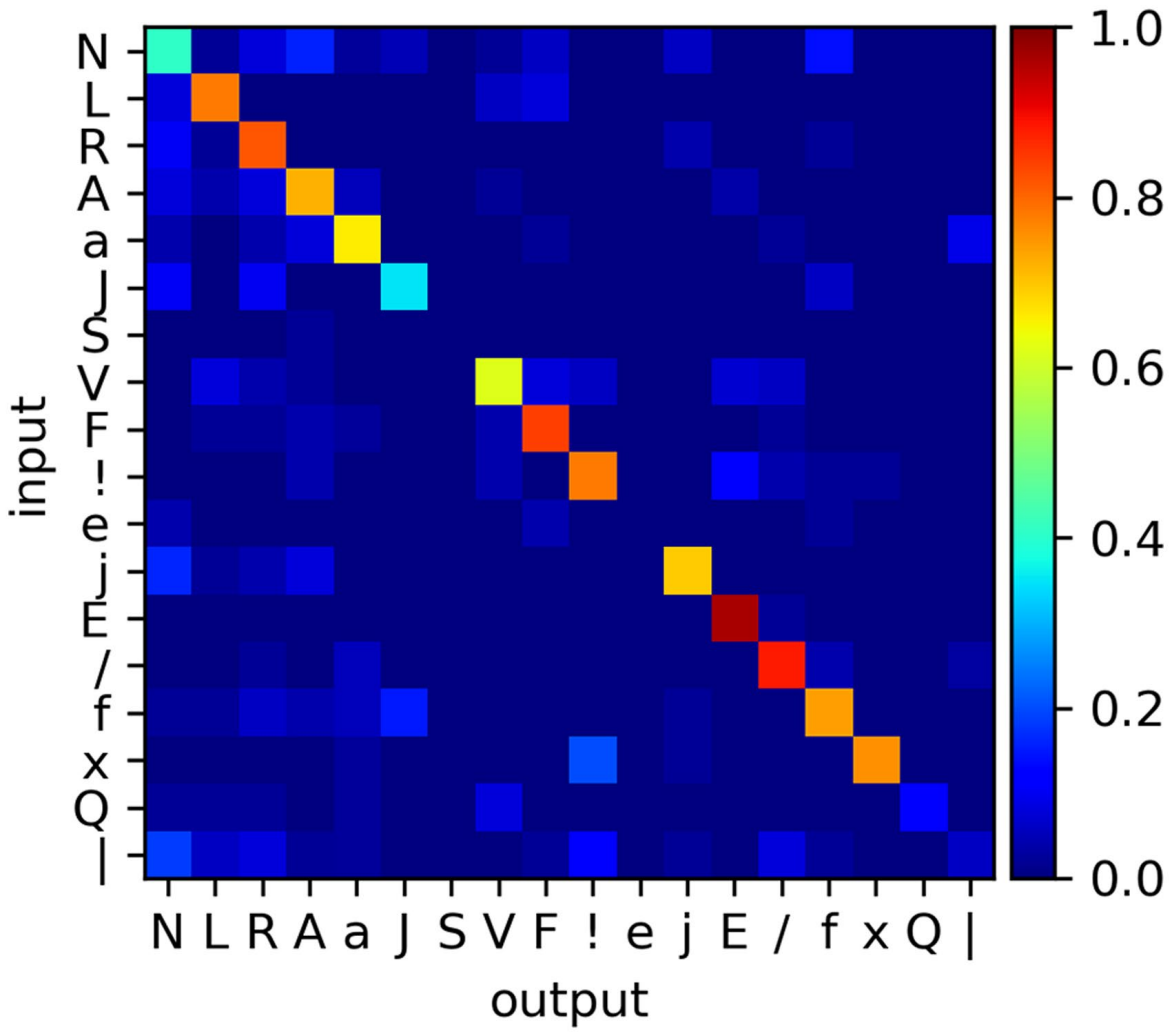
Confusion matrix for the classification task of the MIT-BIH database, after 50 epochs and using a training dataset where each class is capped to a maximum of 500 samples, chosen randomly.

An initial training (50 epochs) is carried out on all the classes (with a maximum of 500 samples per class), to test the capability of the SNN to classify the MIT-BIH data. The test is carried out with a separate set of samples (maximum of 50). We find a post-training accuracy value of 0.683 (the confusion matrix is presented in Figure 11). This is an appreciable result, considering the complexity of the classification task (with a large number of classes, the unbalanced number of samples per class, and the under-dimensioned training); and the rough task design (with no fine-tuning of the SNN design, input signal pre-processing or filtering, absence of credit balancing techniques). This task reveals that the devised system can extrapolate some of the temporal features of the proposed dataset, with potential for further amelioration.

**Figure 12:**
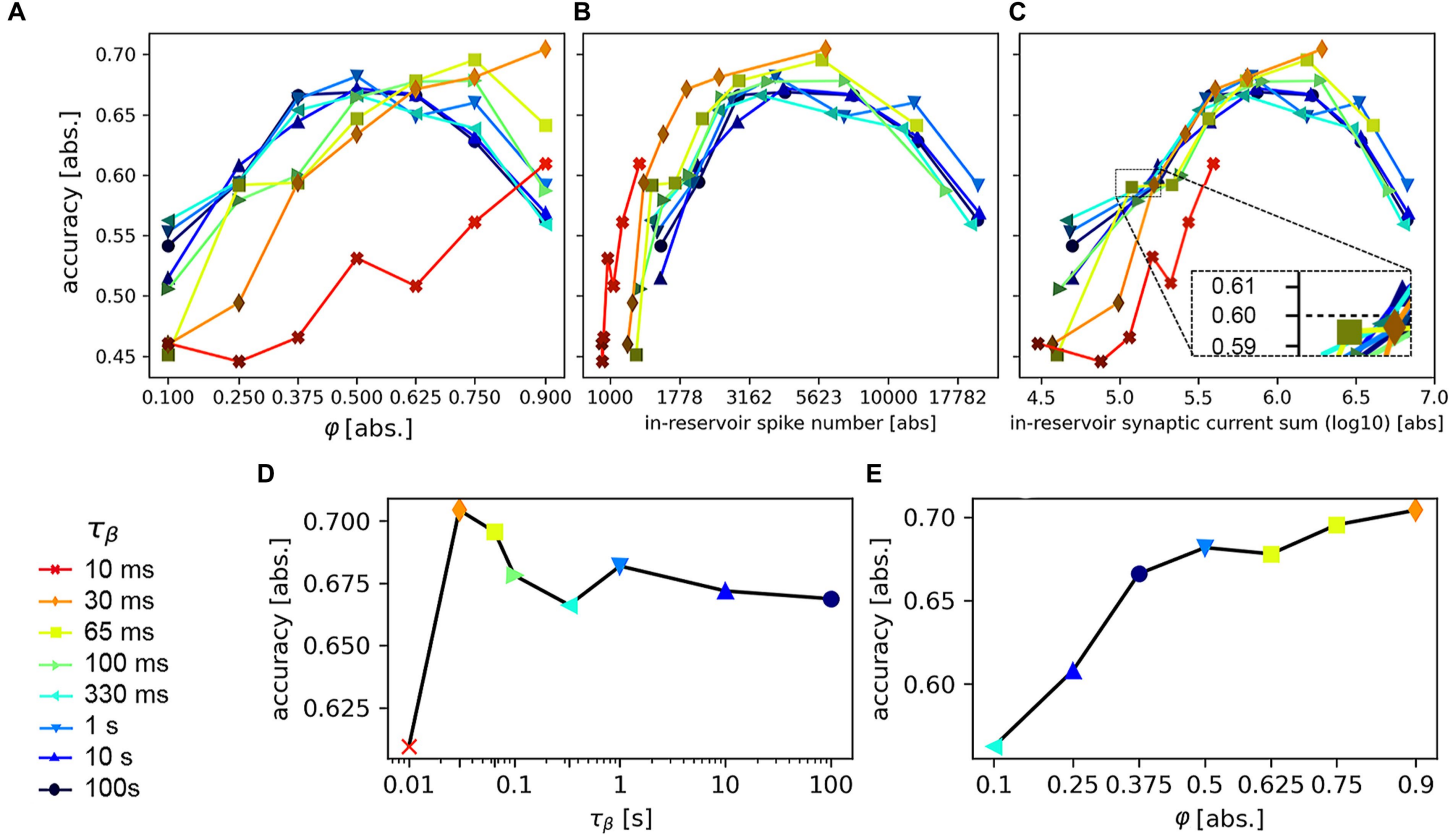
Accuracy results after a 50-epochs training on the recognition of the reduced MIT-BIH dataset, based on the variation of the SNN hyperparameters $\tau_{\beta }$ (color legend on the left) and the scaling factor of the reservoir-to-reservoir synaptic connections, $\varphi_{res}$ . **(A)** Accuracy vs. scaling factor φ ; each line traces the results for a different $\tau_{\beta }$ . **(B)** Accuracy vs. (average) number of reservoir spikes per inference per sample. **(C)** Accuracy vs. (average) integral of the reservoir synaptic current output, per inference per sample. **(D)** Maximum (average) accuracy, as determined from the data shown in **(A)**, as a function of the temporal constant $\tau_{\beta }$ . **(E)** Maximum (average) accuracy as a function of the scaling factor φ .

Equipped with this knowledge, we devise a task that directly explores the causal correlation between accuracy and network design. We simplify the dataset by reducing it to the R, A, V, and F classes, chosen as these count 500 samples, offer relatively high recognition rates, and potentially challenging out-of-diagonal non-zero values (c.f. Figure 11). We train the SNN with the reduced dataset, setting on the system a different combination of $\tau_{\beta }$ (spanning from 10 ms to 100 s) and φ (ranging from 0.1 to 0.9). The post-training data is shown in Figure 12.

This is a basic investigation in the framework of a relevant scientific topic, as the use of multiple timescales in SNN components proved fundamental for efficient data processing (relevant literature is available in Quax et al. (2020)) and, at the same time, difficult to implement in hardware SNNs (MeM-Scales, 2020; Christensen et al., 2022; Jaeger and Catthoor, 2023). Recently, innovative circuit designs have been proposed to support an extension of the temporal dynamics for neurons and synapses (see e.g. Rubino et al., 2021). Additionally, the engineering of the dynamics of solid-state devices like volatile memristors or other technologies (Milo et al., 2020; Demirag et al., 2021; Brivio et al., 2022), have been investigated as tools to implement tunable dynamical elements in SNNs (see e.g., MeM-Scales, 2020; Beilliard and Alibart, 2021; Sarwat et al., 2022). For this simulated task, it is possible to envision a standard CMOS-based hardware platform, which supports the seconds-long LIF neuron temporal dynamics here investigated by means of the solution proposed in Rubino et al. (2021).

Figure 12A reports the (averaged) accuracy values as a function of φ ; each line represents the data obtained using a different temporal constant $\tau_{\beta }$ . This plot evidences that for any given value $\tau_{\beta }$ it is possible to find an optimal value φ (excluding the 10 ms and 30 ms cases, for which we are allowed to infer that the explored φ range would need an extension). The results confirm that fine-tuning of the proposed hyperparameters is conducive to the best performance; and that, generalizing, one can compensate for the limitation imposed by an architectural parameter by adjusting the remaining ones. However, this plot does not evidence yet the causal correlation between the investigated parameters and classification accuracy.

In Figure 12B we plot the same results as a function of the averaged number of spikes, per sample, counted within the reservoir neurons. Here, the results obtained for each temporal constant seem to align and position the peak accuracy values within a range of spikes between 3,000 and 6,000 counts. This result evidences that the highest accuracy can be here attained whenever the reservoir fire rate is adjusted towards an optimal range, below and above which the LSM decreases trainability or fails to extract meaningful higher-dimensional features. Whilst this result does not quantify the optimal hyperparameter combination, it evidences the importance of maintaining a suitable reservoir spiking activity, which finds support in theoretical studies on the subject (Legenstein and Maass, 2007).

Nevertheless, we note that an increase in either the scaling factor φ or the number of pulses engenders a proportional energy cost, which is a technologically-detrimental element in hardware systems. In Figure 12C we plot the same accuracy values, here as a function of the averaged integral of the reservoir synaptic currents, per sample. Indeed, our results correlate higher accuracy and increased energy requirement. This analysis may be used to set an acceptable accuracy as a threshold value, to then determine the system parameters conducing to the best energy performance. For sake of argument, we determine the accuracy of 0.6 as the acceptability limit. Consequently, we find that low $\tau_{\beta }$ values reach such threshold at a generally higher current contribution (see inset in Figure 12C), deeming the “slower” neurons to be the better candidates to limit the energy requirement.

We reserve a last comment for the analysis of the maximal accuracy values (derived from the data shown in Figure 12A), for each of the investigated hyperparameters. We report those against the investigated hyperparameter ranges in Figures 12D,E. Concerning the dependency on the temporal constants $\tau_{\beta }$ we find that low values correlate with higher maximal accuracy (excluding the 10 ms one; it is however plausible to extrapolate the same trend). We notice two outliers, at the 100 ms and 330 ms locations; it is not possible with the data at hand to attribute this feature to statistical variations or the dynamics of the analyzed datasets. However, accuracy reduction as $\tau_{\beta }$ increases seem to lessen at higher $\tau_{\beta }$ values, which implies that very long temporal constants would perform almost identically above the 10 s one (with an accuracy close to 0.67). As for the scaling factor φ , we determine an almost-monotonic increase in the accuracy, which slows just above the accuracy of 0.7 (at φ = 0.9), and that finds a larger drop below the value of φ = 0.375. This result reiterates the considerations extrapolated from the analysis of the data shown in Figures 12B,C: the plot shows that an arbitrary acceptable accuracy can be found only above a scaling factor lower boundary, and that very low power consumption (i.e., low scaling factor) benefits from the use of “slow” neurons.

### 4.4 Summary

We used SHIP to build, simulate, and train arbitrarily-generated SNNs in plausible test cases.

We initially demonstrated the practical use of the platform for a simple task, in which we detailed the construction of the network, its manipulation, and the result retrieval. The training of different networks has been successfully demonstrated, using the FreeSpokenDigit, the Braille, and the MIT-BIH arrhythmia dataset. For each case, we detailed the dataset encoding, the network definition, the training process, and the obtained results. SHIP derived these results by interfacing the user with the PyTorch routines, leveraging the surrogated gradient technique presented in Neftci et al. (2019).

In Section 4.2 we briefly analyzed the post-training behavior of the network, concerning the weight quantization and drift due to an assumed memristive storage technology. An in-depth analysis of the post-training network behavior has been carried out with the MIT-BIH dataset in Section 4.3. The network behavior has been measured after variation of a selection of network hyperparameters finding plausible physical homologues. We have determined the correlation between the potential trained network performance and the network configuration, which exemplifies the use of SHIP as a useful tool for network prototyping.

## 5 Discussion and conclusion

With this manuscript, we summarized the various challenges that the simulation of SNN systems poses, which in turn urged the scientific community to create a wide range of numerical tools, each with its unique key characteristics and advantages, due to the theoretical and computational limitations barring one to access to a one-fit-all solution. This, however, engenders a dichotomy in the numerical tool panorama, where most of the proposed solutions are oriented towards model-driven or data-driven approaches; even in the case of software interfaces intended to fulfill a wide-ranging set of requirements, it often becomes difficult to use one tool to prototype and simulate bespoke models with low effort, even more so if conventional machine-learning techniques are deemed necessary.

With SHIP, we targeted an audience not necessarily trained or familiar with the SNN simulation primitives, but requiring a simple tool to rapidly prototype potential SNN architectures or artificial neuronal/synaptic component functionalities, by way of compact models. We previously identified a few key characteristics that may be beneficial to the end user, and we delivered an environment specifically tailored to attain these.

*An easy-to-learn interface.* SHIP relies on a straightforwardly structured set of instructions, which one can rapidly understand and amend for any arbitrary scope. Network building, inference, and training are performed by way of an easily-interpretable language.

*Low memory and computational requirement*. In SHIP, many of the algorithmic adaptations and design choices shrink the calculation and memory requirement to the bare essential, e.g., the precalculation of the temporal dependencies within the model, the exploitation of PyTorch parallel calculation features, avoidance of repeated memory access, or the optimization of the algorithm handling the SNN data flow. However, we note that properly estimating the optimization reach of our algorithm is a difficult task. In Supplementary material (Section 5) we attempt to compare the performance of SHIP and alternative simulation platforms on the same simulated network. The results indicate that SHIP, as intended, does not add any significant computational burden in the simulation of small networks. We underline that SHIP grants so, yet retaining an essential flexibility of use (for any arbitrary SNN architecture) and modularity (as any model can be developed and employed as-is with no further tailoring required).

*Facilitated development and deployment of user-defined models.* SHIP proposes the simulation of the SNN by use of models fitting the group definition. The underpinning equations fit a very clear and well-structured set of classes’ methods, which one can rapidly understand and amend for their own scopes. Whilst this is certainly not a unique solution amongst the wide panoramic of available simulation platforms, we consistently find that the simplicity of the model development is limited to model-driven platforms, not allowing to rapidly interface with high-throughput-supervised learning routines. This instead remains a key feature of SHIP. Furthermore, to increase interoperability with the already available model frameworks, the development of a PyNN model compiler is under evaluation for future releases, which would also automate the determination of the time-step dependencies (a task now delegated to the user by way of the time_dep method).

*Facilitated access to a wide range of time-dependent parameters and results*. We find that rapid model-building (using natural language) is a considerably useful feature, especially in combination with the ability to monitor any of the states and outputs of the models during inference. This endows the user with an immediate view of the model behavior, helpful during both prototyping and model analysis.

*Methods facilitating the network synaptic weights training*. As shown in Sections 4.2 and 4.3, SHIP natively combines the functionality of SNN simulation with PyTorch training routines, by use of the surrogate gradient technique. Other methodologies, such as on-line training via *ad-hoc* learning rules, may indeed be coded-in whenever required by the end-user by way of bespoke component models.

*Suitability to perform parameter-dependent simulations*. SHIP can treat any of the arguments provided during the SNN building stage as functions. This allows one to rapidly generate arbitrary distributions spanning any spatial coordinate and/or vary along the number of parallel calculations, bestowing the user with a powerful syntax useful for both SNN building (e.g., generating weight distributions at a glance), and inference for an arbitrarily-determined range of SNNs, as shown in Section 4.1. This feature meets our requirement for a platform helping to perform parameter-dependent simulations.

It is necessary to remark on the limitations and computational requirements of the current version of SHIP. As discussed in Section 2, a single tool can not efficiently address any arbitrary task, and the simulation algorithm needs fine-tuning for each scope. SHIP has been designed for the simulation of small networks of custom models and/or arbitrary connectome, for a reduced number of time-steps, on general-purpose workstations. This task is best addressed with a clock-driven algorithm that drives a set of time-discrete models, interoperating in a directed (though streamlined) fashion, to limit unessential operations. Our numerical evaluation demonstrates that, in fact, this framework is highly optimized for the intended objective (see Supplementary material, Section 5). Of course, as a drawback, SHIP is less than optimal for other case scenarios, with two in particular being least suitable: SHIP performs poorly as the number of time-steps, or the number of network parameters, increases. This is due to how SHIP mandates the calculation of the evolution equation on all units, at all time-steps (due to the fixed time-step CD algorithm). SHIP currently does not accelerate through time when and where the absence of spikes would in principle take advantage of a time-skipping technique, unlike platforms reliant on variable time-step or ED algorithms. Another correlated drawback is identified in the choice of the time-step size. It must resolve the fastest dynamics in the simulated system, or be tuned to fit issues arising from the numerical stability of the employed models. However, doing so may lead to oversampling of models that do not require comparatively high temporal resolution. Lastly, we need to mention how the model definition in SHIP (as in all platforms based on PyTorch) relies upon the competence of the user in writing a set of time-discrete equations. This is not a trivial task, especially for complex models (reason for which a PyNN model compiler is being planned).

We eventually refer to the simulation tasks shown in Section 4, which illustrate the use of SHIP in potential case scenarios. The illustrated cases progressively demonstrate how SHIP can be used to (i) prototype a potential SNN, (ii) monitor its internal variables, (iii) simulate inference (iv), train its parameters via surrogate-gradient-enabled conventional machine learning techniques, and eventually (v) combine training and inference-based variable monitoring, to assess a candidate SNN structure or constitutive circuit blocks in terms of classification accuracy and other performance. We have shown how SHIP can indeed fulfill the intended requirement of a simple, flexible tool that experimenters dedicated to hardware-oriented SNN simulations can use for a wide range of tasks and investigations.

## Data availability statement

Publicly available datasets were analyzed in this study. This data can be found here: Jakobovski/free-spoken-digit-dataset: v1. 0.8, available at: https://zenodo.org/record/1342401; Braille Character Dataset, available at: https://www.kaggle.com/datasets/shanks0465/braille-character-dataset; MIT-BIH Arrhythmia Database, available at: https://doi.org/10.13026/C2F305.

## Author contributions

EG: Conceptualization, Formal analysis, Investigation, Methodology, Software, Validation, Visualization, Writing – original draft, Writing – review & editing, Project administration. SS: Conceptualization, Funding acquisition, Project administration, Resources, Supervision, Writing – review & editing. SB: Conceptualization, Formal analysis, Methodology, Project administration, Supervision, Writing – review & editing.
