# Supplementary material for: SHIP: a computational framework for simulating and validating novel technologies in hardware spiking neural networks
Source: Front Neurosci. 2024 Jan 8;17:1270090. doi: 10.3389/fnins.2023.1270090 (PMC10804805; doi:10.3389/fnins.2023.1270090)
Supplement: Supplementary file 1 [file Presentation_1.pdf]

Supplementary Material

SHIP: a computational framework for simulating and validating novel technologies in hardware spiking neural networks

Emanuele Gemo[1*], Sabina Spiga[1], Stefano Brivio[1]

[1]CNR–IMM, Unit of Agrate Brianza, via C. Olivetti 2, Agrate Brianza, Italy

***Correspondence:** Emanuele Gemo: emanuele.gemo@mdm.imm.cnr.it

1 Available emulation platforms

In the table below, we report a non-exhaustive list of the numerical tools that can be directly employed or adapted to the simulation of Spiking Neural Networks (SNNs). This table collects the numerical tools available at the date of writing, limited to the open source ones that offer a maintained or at least non-deprecated version. It details 37 numerical tools, added to the one here illustrated. The table sorts the entries by year of publication of the first release. A legend is provided in caption.

Name	*Year of 1st release*	*Summary of the key characteristics*	*Philosophy*	*Model*	*Temporal progress*	*Language environment*	*GPU / distributed computing*
**GENESIS** (Bower and Beeman, 2007)	1988	It is devised to emulate biological neurons and networks with high resolution; it uses empirical models of neurons and synapses, and it enables individually configurable parameters and connectivity.	MD	DIF.	m-CD	GENESIS Script	DIS. (Crone et al., 2019)
**XPPAUT** (Bard, 1996)	1996	Originally devised as a general-purpose numerical tool for simulating and analyzing dynamical systems, it can also be used for small-sized SNNs.	MD	DIF.	(f,v)-CD	Stand-alone program; C libraries can improve handling or performance	CPU only*
**NEURON** (Hines et al., 2020)	1997	Similar in philosophy to GENESIS.	MD	DIF.	(f,v)-CD	HOC, Python	GPU, (Migliore et al., 2006), DIS.

**NCS** (Drewes, 2005; Hoang et al., 2013)	2000 (ver. 1)	Parallel computation-centered SNN simulator, proposed to tackle the emulation of simplified (single-compartment neuronal models, templated behavioral patterns, etc.), yet realistic, biological systems. Brainlab (Drewes et al., 2009) adds a more user-friendly Python interface.	MD	TD	f*-CD	C/C++, Python	GPU/ DIS.
**EDLUT** (Ros et al., 2006)	2006	It tackles the emulation of biological/biologically inspired networks via the use of look-up tables, possibly generated by external solvers (Carrillo et al., 2007), reducing the otherwise costly solution of the differential equation systems of the (available) neurons' models	MD	DIF.	CD/ ED	C/C++, Python	GPU (CD; ED algorithm works on CPU only)
**NEST** (Gewaltig and Diesmann, 2007)	2007	General purpose emulator, best addressing the analysis of the network's dependency on neuron/synapse models and architectural parameters	MD/ DD	model dependent	v*-CD	SLI, C/C++, Python	DIS. (Ippen et al., 2017)
**CARLSim** (Niedermeier et al., 2022)	2009	Similar in philosophy to NEST, it natively supports GPU-accelerated calculations	MD	DIF.	v-CD	C/C++, Python	GPU
**NeMo** (Fidjeland et al., 2009)	2009	A CUDA-based platform for emulation of large biological networks, that is focused on using the Izhikevich neuron model for large but sparsely connected networks.	MD	TD*	m-CD	C/C++, Python, MATLAB	GPU
**CNS** (Poggio et al., 2010)	2010	Data-driven analysis tool with model-driven elements; CNS is a parallel-computing oriented platform, proposed for large networks, including biological (Hodgkin-Huxley model based) ones. It can handle the neuron connectivity automatically, by way of spatial coordinates.	MD/ DD	model dependent*	f*-CD	C/C++, MATLAB	GPU
**GeNN** (Yavuz et al., 2016)	2011	It proposes a library to generate efficient CUDA code from a simplified custom model building interface, intended to accelerate the emulation of biological-oriented SNNs.	MD	model dependent	f*-CD	C/C++, CUDA, Python	GPU

**N2D2** (Bichler et al., 2017)	2013	It's been purposely developed to design and emulate deep neural networks, in particular dedicated to the integration in embedded systems. It can be adapted to emulate inference and local-rule-based training of spiking networks, see e.g. (Brivio et al., 2021)	MD/ DD	model dependent	dependent on the implementation	C/C++	GPU
**Nengo** (Bekolay et al., 2014)	2014	Conceptually similar to PyNN, it proposes a simplified environment to generate and simulate large-scale networks based on available or user-defined neuron and synapse models. NengoDL (Rasmussen, 2019) is a dedicated fork to the integration of deep learning techniques in Nengo (derived from TensorFlow)	MD/ DD	TD	(f,v)-CD	Python	GPU (Rasmussen, 2019)
**Auryn** (Zenke and Gerstner, 2014)	2014	Simulation environment focused on studying plastic spiking recurrent neural networks of small-to-medium sizes.	MD	TD	Hybrid (f-CD, with ED weight updates)	C/C++	DIS.
**Brian 2** (Stimberg et al., 2019)	2014 (ver. 1)	General purpose environment, that provides tools to i) generate neurons and synapse models via defining a system of differential equations, and ii) to arrange such devices and analyze the emerging network behavior.	MD	DIF.	f-CD (ED available when using linear equations)	Python	GPU (dependent on exteral solvers ses (Stimberg et al., 2020; Alevi et al., 2022))
**NEVESIM** (Pecevski et al., 2014)	2014	Proposes a simplified environment for the construction, simulation and analysis of neural models.	MD	TD	ED (CD is allowed)	C/C++, Python	CPU-only
**ANNarchy** (Vitay et al., 2015)	2015	It draws elements from the PyNN environment interface and the Brian model definition interface. It allows one to efficiently emulate spiking networks and their training via STP (short-term plasticity) or STDP (spike-timing dependent plasticity)	MD	DIF.	f-CD	Python, C/C++	GPU

**MegaSim** (Stromatias et al., 2017)	2016	Tool addressing the event-driven simulation of hardware systems, strongly addressing the simulator performance. It relies on a strongly modular and compartmentalized vision of the modeled components. STDP-learning rules and vision-dataset-oriented modules are available.	MD/ DD	TD	ED (though a clock module can be included)	C/C++	CPU only
**BindsNET** (Hazan et al., 2018)	2018	Environment that is dependent on the PyTorch datatypes, which are used as the mathematical framework to define models for neurons, synapses, and learning rules.	DD	TD	f-CD	Python (PyTorch)	GPU
**DynaSim** (Sherfey et al., 2018)	2018	It enables defining of new neurons and synapse models either via their differential equation systems or via the combining of already-available models. It natively supports tools to post-process the emulated network results, e.g. investigate how the simulated network behavior varies according to predetermined architectural parameters.	MD	DIF.	(f,v)-CD	MATLAB	DIS.
**SPIKE** (Ahmad et al., 2018)	2018	Simulation-speed optimized, GPU-enabled, SNN emulator for bio-inspired network analysis, that provides few commonly adopted neuron, synapse, and learning rule models.	MD	TD	f-CD (a timestep grouping technique is here proposed)	C/C++, CUDA	GPU
**LSNN** (Bellec et al., 2018)	2018	Set of Tensorflow-based libraries adapted to the emulation and training of recurrent SNNs, based on an adaptive leaky integrate and fire model.	MD/ DD	TD	f-CD	Python (Tensorflow)	GPU
**cuSNN** (Paredes-Valles et al., 2020)	2018	Environment defining a small selection of learning rules, neuron or synapse models and layers, which combined enable one with the GPU-accelerated emulation of large SNNs	MD/ DD	TD	f-CD	C/C++, CUDA	GPU

**Slayer** (Shrestha and Orchard, 2018)	2018	Framework centered on the data-driven emulation of SNNs and their synaptic weight/axon delay training based on a modified error backpropagation technique. Currently being integrated into a more comprehensive set of functionalities for the Lava environment (Richter et al., 2021).	DD	TD	f*-CD	C/C++, CUDA, Python	GPU
**RockPool** (Muir et al., 2019)	2019	A platform aimed to bridge the gap between the design and emulation of a spiking network (here viewed as a more general dynamical system), and training of such network by use of deep-learning methods. It is suited to designing SNN hardware specifically. Models of devices, subnetworks, and learning methods can be defined or imported from other platforms (e.g. PyTorch, Jax (Frostig et al., 2018), Brian 2)	MD/ DD	TD/DIF (as it includes Brian2 and Jax model support)	f-CD	Python (PyTorch is supported)	GPU
**SpykeTorch** (Mozafari et al., 2019)	2019	Pytorch extension optimized towards the emulation of STDP and R-STDP feed-forward SNNs using the time-to-first-spike data encoding.	DD	TD	f-CD	Python (PyTorch)	GPU
**PySNN** (Büller, 2020)	2019	Another take on integrating PyTorch with SNN emulation functionalities, comprising a set of local learning rules.	DD	TD	f-CD	Python (PyTorch)	GPU
**s2net** (Zimmer et al., 2019)	2019	PyTorch implementation of dense and convolutional SNN LIF (Leaky Integrate and Fire) layers, reliant on the surrogate gradient-based (Neftci et al., 2019) conventional DNN training methods	DD	TD	f-CD	Python (PyTorch)	GPU
**sinabs** (Lenz and Sheik, 2020)	2019	Similarly to RockPool, this framework is intended to aid the design and deployment of SNNs, with a narrower focus on vision datasets and broader reliance on the PyTorch syntax and its DNN learning routines.	DD	TD	f-CD	Python (PyTorch)	GPU
**DECOLLE** (Kaiser et al., 2020)	2020	A narrow-focused framework that uses PyTorch libraries to implement both SNN emulation functionalities and the proposed DEep COntinuous Local Learning (DECOLLE) rule.	MD/ DD	TD	f-CD	Python (PyTorch)	GPU

**Spice** (Bautembach et al., 2020)	2020	GPU-reliant, clock-driven SNN simulation approach, oriented towards increasing the performance metrics (limited to a single workstation)	DD	TD	f-CD (using timestep grouping as in SPIKE)	C/C++, CUDA	GPU
**Spiking Jelly** (Fang et al., 2020)	2020	A comprehensive set of libraries expanding PyTorch with the SNN emulation functionality, along with data handling and conversion.	DD	TD	f-CD	Python (PyTorch)	GPU
**Sapicore** (Moyal et al., 2021)	2021	Minimal environment reliant on a PyTorch computational back-end. Similarly to SHIP, it's been primarily developed with the view to facilitate the modeling of neuromorphic elements (and their integration with PyTorch), by way of a set of high-level functions and classes.	MD/ DD	Model dependent	Model dependent	Python (PyTorch)	GPU (potential; dependent on the user implementation)
**Norse** (Pehle and Pedersen, 2021)	2021	PyTorch extension, oriented to gather the results of simulated SNNs inference and bio-inspired learning processes; it has been designed with a strong orientation towards sparse computing techniques to better interface with event-based inputs.	MD/ DD	TD	f-CD	Python (PyTorch)	GPU
**Lava** (Richter et al., 2021)	2021	Environment enabling one to design and test SNN hardware with an application-oriented philosophy, providing several neuron models, tools aiding the definition of network topologies, and IO circuitry. It can natively run simulations on CPUs, GPUs, and neuromorphic hardware.	MD/ DD	TD	Hybrid (ED message parsing, m-CD model evaluation)	Python	GPU
**snnTorch** (Eshraghian et al., 2021)	2021	An alternative implementation of spiking layer functionalities to be employed within the PyTorch environment, comprehensive of a set of surrogate gradient functions to enable DNN learning techniques.	DD	TD	f-CD	Python (PyTorch)	GPU
**EvtSNN** (Mo and Tao, 2022)	2022	Based on a previous release, the EDHA simulator (Mo et al., 2021). EvtSNN is an improved version of an event-driven simulator; it is optimized for the training of LIF-based networks by use of the STDP learning rule.	MD	TD	ED	Java, C/C++	CPU only

**Doryta** (Cruz-Camacho et al., 2022)	2022	Successor of NeMo (Plagge et al., 2018); Doryta has been developed to enable one with both simulation of hardware-based SNNs (by way of simplified models), and analysis of the results to gather a few potential performance metrics.	MD/ DD	TD	f*-CD/ ED (only where neurons have positive leak values)	C/C++	DIS.
**SHIP** (this work)	2023	SHIP has been developed to facilitate the simulation of hardware SNN systems, by way of compact models. It fosters features suited for the analysis and performance assessment of the emulated architectures, also integrating PyTorch machine-learning techniques, currently leveraging the surrogate gradient technique (Neftci et al., 2019)	MD/ DD	TD	(f,m)-CD (multiple time-step sizes can be predetermined to carry out the simulation task)	Python (PyTorch)	GPU

Table 1. Panoramic of the available open-source numerical tools enabling one to carry out emulation of hardware-based SNN systems. Legend: MD – model-driven approach; DD – data-driven approach; TD – time-discrete equation system model; DIF. – differential equation system model; CD – clock-driven temporal handling (f- fixed, v- variable, m- multi-clock); ED – event-driven temporal handling; DIS. – distributed computing available. *Data unclear or missing in the available references; tabulated data interpreted from the source code.

2 Group sorting algorithm

Here we provide few more details to explain the rationale behind the objective function of the proposed sorting algorithm, and its practical functioning.

Objective function: reduction of the delay-substituted DAM LTS lower triangular sum

The algorithm's main goal is to find the group order so that, during the simulation, it lowers the count of events breaking the causal correlation or inducing computational artifacts (due to the non-negligible effect of the time-step size). For feedforward networks the task is trivial as the groups are inherently hierarchically-sorted (each finds a single source, and a single target). However, the same task is more difficult for recurrent networks, in which groups may find multiple sources and targets.

To define a unique method to efficiently linearize arbitrarily-complex networks, we evaluated the hypothetical edge scenarios for which a candidate solution consistently leads to the most suitable sorting orders. We have found that the best solution leverages the delay time values, decreasing the total of the negative-valued delays. This approach directly minimizes the count of possible artifacts, in which groups “hang” waiting for a late, expected input.

Here follows a practical example to show the potential effect of an arbitrary, un-mediated linearization. Let’s assume a circularly connected network AB, with delay times AB = 0 and BA =1, simulated using timesteps of size 1. Each group relays any input to the connected ones (as in the case of a low-threshold LIF neuron). We omit discussing the synaptic connections for sake of simplicity, integrating the delay

effect in the A and B groups. We start with a signal being delivered from A (noting that an analogous version of the events happens when starting with B).

The network linearized as AB (LTS of 0) behaves as follows (a picture is found in Figure S1):

- at t=0, the signal travels from A to B, is received from B, and sent back to A (with a delay of 1)
- at t=1, A receives the delayed signal from B and sends it back to B; B receives the signal and sends it to A (with a delay, not received)
- at t>1, the chain of events follows the one of t=1; the network "spikes" at a frequency of 2 relays per timestep.

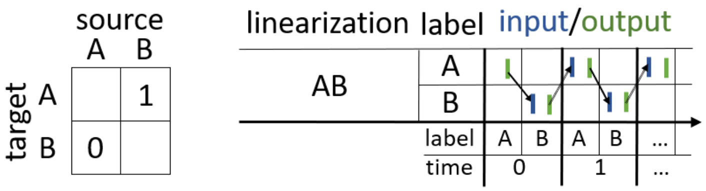

Figure S1. Behavior of the ideal circularly-connected AB network, linearized as A-B. Left: delay-substituted DAM. Right: schematic representation of the system temporal evolution. Data transfer is depicted using black arrow (solid for zero-valued delay, double-lined for 1-valued delays).

The network linearized as BA (LTS of 1) sees the following (a picture is reported in Figure S2):

- at t=0, the signal travels from A to B (not received)
- at t=1, B receives the signal from A (with an artifact delay of 1, tracked as a red arrow in Figure S2), and sends it back to A with a delay of 1 (A does not receive the signal)
- at t>1, the chain of events follows the one of t=1; the network "spikes" at a frequency of 1 relay per timestep, halved with respect to the ideal behavior.

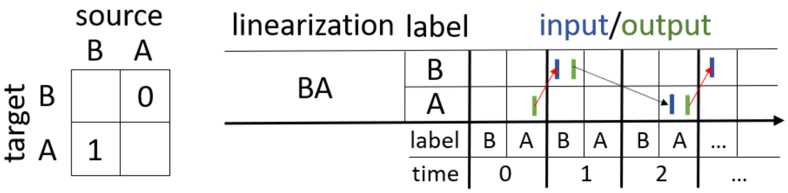

Figure S2. Behavior of the ideal circularly-connected AB network, linearized as B-A. Left: delay-substituted DAM. Right: schematic representation of the system temporal evolution. Data transfer is depicted using black arrow (solid for zero-valued delay, double-lined for 1-valued delays). Red arrows track the computational artifact events.

The two linearizations yield different outputs, with the former correctly reproducing the continuous-time representation of the circular system; and the latter not merely being shifted in time, but seeing an effective reduction by half of the relaying frequency, due to the computational artifacts (red arrows). Whilst simplistic, this example is effective in demonstrating the main practical issues arising from a non-mediated linearization of the system.

Sorting algorithm functioning/computational requirement

We here summarize the algorithm's practical functioning. Given N source-target group pairs, the algorithm performs the following:

i) calculates all potential contributions to the LTS for all N pairs,
ii) calculates the LTS for all N^2 arrangement and swaps of the pairs,
iii) sorts the N^2-wide list of sequences (by increasing LTS value); and only then,
iv) progressively evaluates each sequence (with at most N sums and logical statement evaluations) until finding the earliest feasible one.

We reiterate that the low-LTS sequence is used to set the IO addresses of each group, thus the calculation is performed once.

Strategies are put in place to avoid the evaluation of the whole N-long sequence, if unfeasible elements within each sequence are identified (e.g. having A before B, B before C, and then C before A). Even assuming N>100 (which is unlikely given the scope of the platform), the worst-case scenarios would reasonably see the algorithm carrying out less than a hundred sums and logical statement evaluations, for a few tens of thousands of sequences. The total runtime of this algorithm is likely within a single second, even with dated hardware. In more practical scenarios, the computational impact of this algorithm is irrelevant.

3 SHIP interface: an explained practical example

For sake of completeness, we report the code that generates the data shown in Figure 8b of the main article. This piece of code hopefully exemplifies the main features of SHIP interface, and the potential results that can be attainable through its use. We then use this example to further demonstrate an useful feature embedded within the SHIP interface, which allows one to use a synthetic notation to state a wide range of value distributions (as arguments of each group instation).

The code generating the data in Figure 8b is as follows:

```
# import classes and functions
from SHIP import (network, # network class
                  inputN, # input neuron class
                  lS_1o, # 1st order leaky synapse class
                  lifN, # LIF neuron class
                  refractory) # refractory superclass
from torch import (manual_seed,
                   rand,
                   arange,
                   zeros,
                   normal)
# preliminary ops, determining emulated time and other minor details
eps = 1e-6 #small number
batch_size = 10 #number of parallel simulations
time, dt = .1, 1e-4 #emulated time [seconds], time-step size [seconds]
nts = int(time//dt + time%dt) #number of time-steps
ns = [3,1] #network neuron layer size
```

```
# input generation
rate = 20 #[Hz]
manual_seed(3000)
poisson_input = (rand(nts,ns[0])<(rate*dt)).expand(batch_size,nts,ns[0])
# define network
snn = network()
# add neuron groups
snn.add(inputN, #add input_neuron group
        'I', #group tag (mandatory)
        N = ns[0]) #number of units within the group
snn.add(refractory(lifN), 'N1', N = ns[1], #add refractory LIF group
        tau_beta = arange(10e-3,100e-3+eps,10e-3).unsqueeze(-1),
       #temporal constant [s]
        thr = 1.,#threhshold potential [a.u]
        _u_ = lambda b,n:
normal(zeros(n),1).abs().unsqueeze(0).expand(b,n),
        #potential at t = 0 [a.u.]
        _u0_ = 0., #rest/reset potential
        refr_time = 10e-3) #refractory time [seconds]
# add synaptic group
snn.add(lS_1o, 'S1', source = 'I', target = 'N1', #add synapse group
        w_scale = 150, #synaptic weight global scaling factor
        tau_alpha = 5e-3, #temporal constant [s]
        delay_time = 0e-3, #delay time
        w__ = rand) # synaptic weight matrix

# init network and monitors
snn.set_monitor(**{'S1':['output','I'],'N1':['u','output']})
snn.init(dt = dt, nts = nts, batch_size = batch_size)
# run simulation and gather data
snn.run(poisson_input) # run emulation
data = snn.get_monitored_results() # gather data
```

Using the script example above, we discuss a practical feature of SHIP which we believe being extremely useful during the network building stage, though too technical to be highlighted within the manuscript. SHIP has the option to parse the arguments of the group class as a generator function, which can be called to yield dynamically-generated data. This feature is helpful for two reasons: i) it allows one to re-generate data according to arbitrary distributions, every time the network is initialized; ii) it also allows one to handle the size of the generated tensors, based on the number of the group's components, and/or the number of the parallel batches. This functionality is provided with subtle changes to the argument names, namely the presence of underscore symbols before and/or after the argument name. Examples of its use are present in the code section reported above, where the arguments `u`, `u0`, and `w` are written respectively as `_u_`, `_u0_`, and `w__`.

The rules applied by the data parser are the following:

1. Underscores before and/or after the argument names instruct SHIP to interpret the data as a generator function.
2. SHIP provides the eventual generator function with specific secondary arguments, determined on the basis of the underscore configuration:
    i. One underscore after the argument name (viz. `arg_`) instructs SHIP to provide the generator function with the number of components (of the group)
    ii. Two underscores after the argument name (viz. `arg__`) provide the argument generator function with the number of components of both (a) the source and (b) target groups
    iii. One underscore before the argument name (viz. `_arg`) provides the argument generator function with the batch size as its argument.
    iv. After- and before- underscore configurations can be combined
3. Depending on the datatype of the argument value, SHIP will perform the following:
    i. A callable (function) argument value is used as is (with the proviso that the callable can accept the number of suggested secondary arguments)
    ii. A numeric argument value is intended to be copied across a tensor, whose size is stated by the provided arguments.

Returning to the previous example, we describe how SHIP reads the user-provided instructions:

a. the argument `u`, expressed as `_u_= lambda b,n: [..]`, tells the data parser that SHIP will use the `lambda` function as is, along with two arguments: the batch-size and number-of-component values
b. the argument `u0`, expressed as `_u0_ = 0.`, tells the data parser to instruct SHIP to generate a bi-dimensional tensor of zeros, of size batch-size, number-of-components of the group
c. the argument `w`, expressed as `w__ = rand`, tells the data parser to instruct SHIP to use the `rand` function, along with the number-of-components of both source and target groups as arguments.

This notation may not be immediately understandable. However, we deem this feature to be highly efficient in setting arbitrarily-generated distributions, onto the parameters of the network. This in turn can be remarkably useful to rapidly vary sets of parameters during the validation stage of the modelled system or to collect results as a function of parameter variations along predetermined parameter ranges.

4 Drift model and calculation of the synaptic parameters

In the circuit design proposed in (Esmanhotto et al., 2022), two memristors regulate the conductance of the inputs of an operational amplifier, whose output would be proportional to the differential reading of the conductance of the memristors (i.e. the operational amplifier is configured as a differential amplifier). In this scheme, drift affects the reading of both inverting and non-inverting inputs.

Our model has been obtained from the experimental data fitting. It proposes a time and set-state dependency of each memristor drift as a normal distribution of mean $\boldsymbol{\mu}$ and standard deviation $\boldsymbol{\sigma}$. Our resulting set of equations is proposed below:

$$\boldsymbol{\mu(t, G_0) = (G_0 < a) \cdot \left((bG_0 - c)log_{10}(t)\ + dG_0 + e\right)\ +\ (G_0 \geq a) \cdot (-log_{10}(t)f + G_0)}$$

$$\boldsymbol{[\sigma/\mu](t, \sigma_0) = (g[\sigma/\mu]_0 - h)log_{10}(t) - i[\sigma/\mu]_0 + j}$$

$$\boldsymbol{[\sigma/\mu]_0(G_0) = k + \frac{l}{G_0 - m}}$$

with the numerical values for each of the symbolic parameters (units in square brackets) shown in Table 2. The parameter $\boldsymbol{G_0}$ refers to the as-written conductance, at t=0.
We note that the above equations reliably reproduce the portrayed experimental data, and allow to interpolate the memristor behavior as a function of time, and the as-written conductance state (at t=0). Due to the fit process and result, this model reliability is comprised between 0.5 and 10000 seconds.

Table 2. Numerical values of the parameters used in our bespoke drift model, derived from the experimental data shown in (Esmanhotto et al., 2022)**.**

$\boldsymbol{a}$ **[μS]**	$\boldsymbol{b}$ **[1]**	$\boldsymbol{c}$ **[μS]**	$\boldsymbol{d}$ **[1]**	$\boldsymbol{e}$ **[μS]**	$\boldsymbol{f}$ **[μS]**	$\boldsymbol{g}$ **[1]**
64.41	0.2302	15.24	0.9546	4.82	0.4129	1.047
$\boldsymbol{h}$ **[1]**	$\boldsymbol{i}$ **[1]**	$\boldsymbol{j}$ **[1]**	$\boldsymbol{k}$ **[1]**	$\boldsymbol{l}$ **[μS]**	$\boldsymbol{m}$ **[μS]**	
0.006294	0.01677	0.005473	0.005127	0.85543	38.72	

In our simulation, we calculate the drift of both inverting and non-inverting memristors, assuming that for the positive (negative) case the memristor located at the non-inverting (inverting) branch is set to the minimal value of 50 μS. A maximal value of 120 μS is also assumed.

To calculate the network parameters as a function of the elapsed time, the as-written network parameters are globally shifted and scaled so that zeros and maximal values match the minimal and maximal conductance values respectively. These values serve as the required $\boldsymbol{G_0}$ parameter. The new conductance is then calculated as the differential contribution between the $\boldsymbol{G(t)}$ values of the inverting and non-inverting channels, randomly generated according to a normal distribution using the mean $\boldsymbol{\mu(t, G_0)}$ and standard deviation $\boldsymbol{\sigma} = \mu(t, G_0) \cdot [\sigma/\mu]\left(t, [\sigma/\mu]_0(G_0)\right)$ yielded by the drift model. The new conductance is then shifted-rescaled into the network weight parameter, using the inverse of the transformation originally used to calculate $\boldsymbol{G_0}$.

5 Algorithm performance optimization

We here explore in a few more detail the computational efficiency of SHIP, which we tuned to be most efficient form moderately-sized networks of arbitrary complexity. For this scope, we can use the benchmark script published by Open Neuromorphic organization (Open Neuromorphic, 2023), which allows us to compare the performance on the same task of several available platforms (we here use RockPool, Sinabs, and snnTorch as comparison standpoints). We remark that the comparison here reported is limited in scope (measuring the numerical optimization of the algorithm for the forward and backward calls on plausible networks), as it merely measures one of the many possible metrics that is unfeasible to collapse into a single value (and is therefore prone to subjectivity (Yik et al., 2023)). We therefore consider the following results as purely indicative.
The benchmark script measures the calculation time of the *Forward* call (i.e. inference simulation, platform-dictated) and the *Backward* call (i.e. gradient calculation). The latter is solved entirely by

PyTorch, but its complexity also depends on the optimization of i) the Forward operation, and ii) the backward function differentiation. The task is carried out for 500 steps on a simple feed-forward network composed of two *N*-sized layers (a linear layer and a LIF neuron layer), end-to-end connected, stimulated by a (externally-generated) Poissonian spiking input. In SHIP, we instate a network using the `inputN`, the `wireS` and the `lifN` classes, which perform an equivalent task as the one proposed in the benchmark. We measure the calculation time for a set of *N* values ranging from 1024 to 16384, so to gather a larger picture of the platform performance based on the network size. This strategy also attempts to widen the otherwise limited scope of the simulation task, which is here restricted to a single case scenario.
The results are shown in Figure S3.

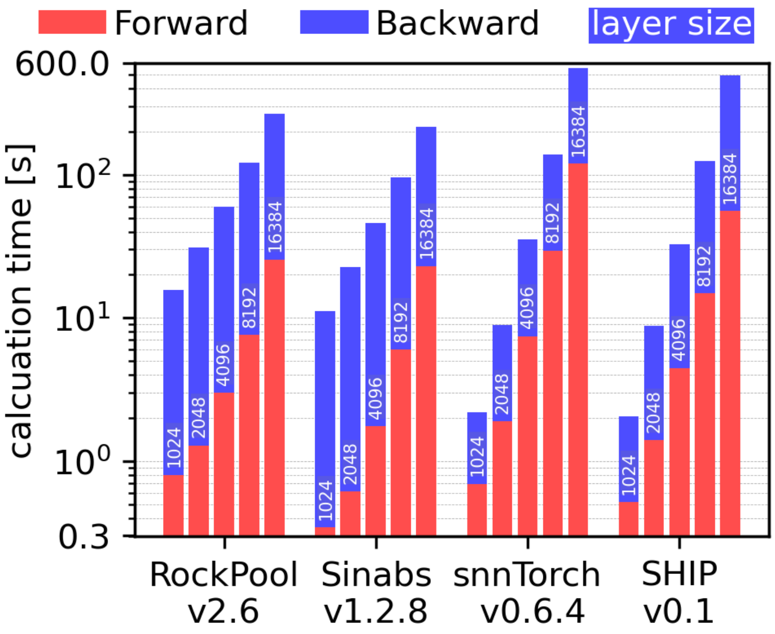

Figure S3**.** Calculation time for the forward (red) and backward (blue) pass of several platforms (RockPool, Sinabs, snnTorch, SHIP) as a function of the network size (ranging from 1024 to 16384; indicated along the plotted bars). The performance has been measured by way of the benchmark script proposed by the Open Neuromorphic organization (Open Neuromorphic, 2023).

Figure S3 plots the forward and backward time for each *N*-value/platform (note the log-log scale). The bars are grouped by platform, to ease the reading of the data. The results we gathered show that all platforms see an increase in the calculation time as the network size grows. In particular for SHIP, we note that it has a relatively low gross calculation time, up to and including the *N* value of 4096 neurons. Additionally, like snnTorch, the Backward time results are lower at low *N* values, but scale poorly as the network size increases (which in contrast RockPool and Sinabs manage better). The gathered results demonstrate that SHIP is best optimized for the simulation of the inference and training of small networks.
We also reiterate that the network complexity has a relatively marginal influence on the algorithm performance (see Section 2.1.3 of the manuscript), as the algorithm merely traverses the time axis and group list to carry out the `advance_timestep` method of each group (this strategy removes any further operation dependent on the architecture).

We can therefore derive that SHIP has been optimized as intended. The results we gathered, and the algorithm strategy of choice, indicate that SHIP is suitable for the prototyping task on small networks of arbitrary complexity, on accessible workstations without GPU acceleration.

We further explore the plausible performance of SHIP outside its intended scope, simulating the forward call (inference) on a medium-sized network. We limit the task to the forward operation, on a 5-layer LIF neuron – 1st order leaky synapse network, counting 10^6 parameters, for 1000 time-steps. This task requires ~5.8 seconds to reach completion in SHIP. Identical results are obtained with a single recurrently-connected layer SNN architecture, again counting 10^6 parameters (as expected). An equivalent task is carried out on RockPool, which has been already shown to have a better optimization for larger networks; and on Brian2, a well-documented ODE solver tuned for SNN simulations, chosen as it adopts a different solver approach to both SHIP and RockPool. This task requires ~69.6 seconds to solve in Brian2, and ~1.6 seconds to solve in RockPool. From this result, we infer that SHIP may be still used for medium-sized networks, though one must expect a relatively longer computational time than the one of available alternatives.

We remark that the computational time gap must be read in light of the characteristics of each platform, as discussed extensively in the manuscript. Brian2 can solve very generally-posed problems in the form of ODE systems, enabling one with an unparalleled degree of flexibility. Optimization of this task however is not easily attainable, thus one has to renounce performance. In contrast, RockPool offers numerous modules for fast prototyping of SNNs, but requires case-specific encoding where arbitrary neuronal and synaptic models are employed (especially in the case of recurrent architectures). SHIP offers a solution mid-way in this spectrum; it retains sufficient flexibility of use for the simulation of SNN systems, as its model can be used in a plug-and-play fashion (akin to Brian2, though in SHIP models can not be explicitly set as ODE systems); yet it retains a performance comparable to the one of RockPool.

6 Funding

This work has been funded via the EU-Horizon2020 research project MeM-Scales (www.memscales.eu), grant no. 871371.

7 References

Ahmad, N., Isbister, J. B., Smithe, T. S. C., and Stringer, S. M. (2018). Spike: A GPU Optimised Spiking Neural Network Simulator. *bioRxiv*. doi: 10.1101/461160.

Alevi, D., Stimberg, M., Sprekeler, H., Obermayer, K., and Augustin, M. (2022). Brian2CUDA: Flexible and Efficient Simulation of Spiking Neural Network Models on GPUs. *Front. Neuroinform.* 16, 883700. doi: 10.3389/fninf.2022.883700.

Bard, G. (1996). XPPAUT: X-windows Phase Plane plus Auto. Available at: https://sites.pitt.edu/~phase/bard/bardware/xpp/xpp.html.

Bautembach, D., Oikonomidis, I., Kyriazis, N., and Argyros, A. (2020). Faster and Simpler SNN Simulation with Work Queues. in *2020 International Joint Conference on Neural Networks (IJCNN)* (IEEE), 1–8. doi: 10.1109/IJCNN48605.2020.9206752.

Bekolay, T., Bergstra, J., Hunsberger, E., DeWolf, T., Stewart, T. C., Rasmussen, D., et al. (2014).

Nengo: a Python tool for building large-scale functional brain models. *Front. Neuroinform.* 7, 48. doi: 10.3389/fninf.2013.00048.

Bellec, G., Salaj, D., Subramoney, A., Legenstein, R., and Maass, W. (2018). Long short-term memory and learning-to-learn in networks of spiking neurons. in *Advances in Neural Information Processing Systems 2018*, 787–797. Available at: https://proceedings.neurips.cc/paper_files/paper/2018/file/c203d8a151612acf12457e4d67635a95-Paper.pdf.

Bichler, O., Briand, D., Gacion, V., Bertelone, B., Allenet, T., and Thiele, J. C. (2017). N2D2-neural network design & deployment. Available at: https://cea-list.github.io/N2D2-docs/index.html.

Bower, J. M., and Beeman, D. (2007). "Constructing Realistic Neural Simulations with GENESIS," in *Neuroinformatics. Methods in Molecular Biology™, vol 401* (Humana Press), 103–125. doi: 10.1007/978-1-59745-520-6_7.

Brivio, S., Ly, D. R. B., Vianello, E., and Spiga, S. (2021). Non-linear Memristive Synaptic Dynamics for Efficient Unsupervised Learning in Spiking Neural Networks. *Front. Neurosci.* 15, 580909. doi: 10.3389/fnins.2021.580909.

Büller, M. B. (2020). Supervised Learning in Spiking Neural Networks. Available at: http://resolver.tudelft.nl/uuid:256f7044-862d-4b53-b395-973dadbb7a00.

Carrillo, R. R., Ros, E., Barbour, B., Boucheny, C., and Coenen, O. (2007). Event-driven simulation of neural population synchronization facilitated by electrical coupling. *Biosystems* 87, 275–280. doi: 10.1016/j.biosystems.2006.09.023.

Crone, J. C., Vindiola, M. M., Yu, A. B., Boothe, D. L., Beeman, D., Oie, K. S., et al. (2019). Enabling Large-Scale Simulations With the GENESIS Neuronal Simulator. *Front. Neuroinform.* 13, 69. doi: 10.3389/fninf.2019.00069.

Cruz-Camacho, E., Qian, S., Shukla, A., McGlohon, N., Rakheja, S., and Carothers, C. D. (2022). Evaluating Performance of Spintronics-Based Spiking Neural Network Chips using Parallel Discrete Event Simulation. in *SIGSIM Conference on Principles of Advanced Discrete Simulation* (New York, NY, USA: ACM), 69–80. doi: 10.1145/3518997.3531025.

Drewes, R. (2005). Modeling the brain with NCS and Brainlab. *LINUX J. online*. Available at: https://www.linuxjournal.com/article/8038.

Drewes, R., Zou, Q., and Goodman, P. H. (2009). Brainlab: A Python toolkit to aid in the design, simulation, and analysis of spiking neural networks with the neocortical simulator. *Front. Neuroinform.* 3, 16. doi: 10.3389/neuro.11.016.2009.

Eshraghian, J. K., Ward, M., Neftci, E., Wang, X., Lenz, G., Dwivedi, G., et al. (2021). Training Spiking Neural Networks Using Lessons From Deep Learning. *arXiv Prepr. arXiv2109.12894*.

Esmanhotto, E., Hirtzlin, T., Bonnet, D., Castellani, N., Portal, J.-M., Querlioz, D., et al. (2022). Experimental Demonstration of Multilevel Resistive Random Access Memory Programming for up to Two Months Stable Neural Networks Inference Accuracy. *Adv. Intell. Syst.* 4, 2200145. doi: 10.1002/aisy.202200145.

Fang, W., Chen, Y., Ding, J., Chen, D., Yu, Z., Zhou, H., et al. (2020). SpikingJelly. Available at: https://github.com/fangwei123456/spikingjelly.

Fidjeland, A. K., Roesch, E. B., Shanahan, M. P., and Luk, W. (2009). NeMo: A Platform for Neural Modelling of Spiking Neurons Using GPUs. in *2009 20th IEEE International Conference on Application-specific Systems, Architectures and Processors* (IEEE), 137–144. doi: 10.1109/ASAP.2009.24.

Frostig, R., Johnson, M. J., and Leary, C. (2018). Compiling machine learning programs via high-level tracing. in *Systems for Machine Learning 2018* (SysML). Available at: https://mlsys.org/Conferences/doc/2018/146.pdf.

Gewaltig, M.-O., and Diesmann, M. (2007). NEST (NEural Simulation Tool). *Scholarpedia* 2, 1430. doi: 10.4249/scholarpedia.1430.

Hazan, H., Saunders, D. J., Khan, H., Patel, D., Sanghavi, D. T., Siegelmann, H. T., et al. (2018). BindsNET: A Machine Learning-Oriented Spiking Neural Networks Library in Python. *Front. Neuroinform.* 12, 89. doi: 10.3389/fninf.2018.00089.

Hines, M., Carnevale, T., and McDougal, R. A. (2020). "NEURON Simulation Environment," in *Encyclopedia of Computational Neuroscience* (New York, NY: Springer New York), 1–7. doi: 10.1007/978-1-4614-7320-6_795-2.

Hoang, R. V., Tanna, D., Jayet Bray, L. C., Dascalu, S. M., and Harris, F. C. (2013). A novel CPU/GPU simulation environment for large-scale biologically realistic neural modeling. *Front. Neuroinform.* 7, 19. doi: 10.3389/fninf.2013.00019.

Ippen, T., Eppler, J. M., Plesser, H. E., and Diesmann, M. (2017). Constructing Neuronal Network Models in Massively Parallel Environments. *Front. Neuroinform.* 11, 30. doi: 10.3389/fninf.2017.00030.

Kaiser, J., Mostafa, H., and Neftci, E. (2020). Synaptic Plasticity Dynamics for Deep Continuous Local Learning (DECOLLE). *Front. Neurosci.* 14, 424. doi: 10.3389/fnins.2020.00424.

Lenz, G., and Sheik, S. (2020). SINABS. Available at: https://gitlab.com/aiCTX/sinabs.

Migliore, M., Cannia, C., Lytton, W. W., Markram, H., and Hines, M. L. (2006). Parallel network simulations with NEURON. *J. Comput. Neurosci.* 21, 119–129. doi: 10.1007/s10827-006-7949-5.

Mo, L., Chen, X., and Wang, G. (2021). EDHA: Event-Driven High Accurate Simulator for Spike Neural Networks. *Electronics* 10, 2281. doi: 10.3390/electronics10182281.

Mo, L., and Tao, Z. (2022). EvtSNN: Event-driven SNN simulator optimized by population and pre-filtering. *Front. Neurosci.* 16, 944262. doi: 10.3389/fnins.2022.944262.

Moyal, R., Einhorn, M., Forest, J., Borthakur, A., and Cleland, T. A. (2021). Sapicore. Available at: https://github.com/cplab/sapicore.

Mozafari, M., Ganjtabesh, M., Nowzari-Dalini, A., and Masquelier, T. (2019). SpykeTorch: Efficient Simulation of Convolutional Spiking Neural Networks With at Most One Spike per Neuron. *Front. Neurosci.* 13, 625. doi: 10.3389/fnins.2019.00625.

Muir, D. R., Bauer, F., and Weidel, P. (2019). Rockpool Documentation. doi: 10.5281/zenodo.3773845.

Neftci, E. O., Mostafa, H., and Zenke, F. (2019). Surrogate Gradient Learning in Spiking Neural Networks: Bringing the Power of Gradient-Based Optimization to Spiking Neural Networks. *IEEE Signal Process. Mag.* 36, 51–63. doi: 10.1109/MSP.2019.2931595.

Niedermeier, L., Chen, K., Xing, J., Das, A., Kopsick, J., Scott, E., et al. (2022). CARLsim 6: An Open Source Library for Large-Scale, Biologically Detailed Spiking Neural Network Simulation. in *2022 International Joint Conference on Neural Networks (IJCNN)* (IEEE), 1–10. doi: 10.1109/IJCNN55064.2022.9892644.

Open Neuromorphic (2023). Available at: https://open-neuromorphic.org/.

Paredes-Valles, F., Scheper, K. Y. W., and de Croon, G. C. H. E. (2020). Unsupervised Learning of a Hierarchical Spiking Neural Network for Optical Flow Estimation: From Events to Global Motion Perception. *IEEE Trans. Pattern Anal. Mach. Intell.* 42, 2051–2064. doi: 10.1109/TPAMI.2019.2903179.

Pecevski, D., Kappel, D., and Jonke, Z. (2014). NEVESIM: event-driven neural simulation framework with a Python interface. *Front. Neuroinform.* 8, 70. doi: 10.3389/fninf.2014.00070.

Pehle, C., and Pedersen, J. E. (2021). Norse - A deep learning library for spiking neural networks. doi: 10.5281/zenodo.4422025.

Plagge, M., Carothers, C. D., Gonsiorowski, E., and Mcglohon, N. (2018). NeMo. *ACM Trans. Model. Comput. Simul.* 28, 1–25. doi: 10.1145/3186317.

Poggio, T., Knoblich, U., and Mutch, J. (2010). CNS: a GPU-based framework for simulating cortically-organized networks. Available at: http://hdl.handle.net/1721.1/51839.

Rasmussen, D. (2019). NengoDL: Combining Deep Learning and Neuromorphic Modelling Methods. *Neuroinformatics* 17, 611–628. doi: 10.1007/s12021-019-09424-z.

Richter, M., Williams, M. G. K., Plank, P., Shrestha, S. B., Risbud, S. R., Weidel, P., et al. (2021). Intel’s Neuromorphic Computing Lab. Lava: A Software Framework for Neuromorphic Computing. Available at: https://github.com/lava-nc/lava.

Ros, E., Carrillo, R., Ortigosa, E. M., Barbour, B., and Agís, R. (2006). Event-Driven Simulation Scheme for Spiking Neural Networks Using Lookup Tables to Characterize Neuronal Dynamics. *Neural Comput.* 18, 2959–2993. doi: 10.1162/neco.2006.18.12.2959.

Sherfey, J. S., Soplata, A. E., Ardid, S., Roberts, E. A., Stanley, D. A., Pittman-Polletta, B. R., et al. (2018). DynaSim: A MATLAB Toolbox for Neural Modeling and Simulation. *Front. Neuroinform.* 12, 10. doi: 10.3389/fninf.2018.00010.

Shrestha, S. B., and Orchard, G. (2018). Slayer: Spike layer error reassignment in time. in *Advances in Neural Information Processing Systems 2018*, 31. Available at: https://proceedings.neurips.cc/paper_files/paper/2018/file/82f2b308c3b01637c607ce05f52a2fed-Paper.pdf.

Stimberg, M., Brette, R., and Goodman, D. F. (2019). Brian 2, an intuitive and efficient neural simulator. *Elife* 8, e47314. doi: 10.7554/eLife.47314.

Stimberg, M., Goodman, D. F. M., and Nowotny, T. (2020). Brian2GeNN: accelerating spiking neural network simulations with graphics hardware. *Sci. Rep.* 10, 410. doi: 10.1038/s41598-019-54957-7.

Stromatias, E., Soto, M., Serrano-Gotarredona, T., and Linares-Barranco, B. (2017). An Event-Driven Classifier for Spiking Neural Networks Fed with Synthetic or Dynamic Vision Sensor Data. *Front. Neurosci.* 11, 350. doi: 10.3389/fnins.2017.00350.

Vitay, J., Dinkelbach, H. Ü., and Hamker, F. H. (2015). ANNarchy: a code generation approach to neural simulations on parallel hardware. *Front. Neuroinform.* 9, 19. doi: 10.3389/fninf.2015.00019.

Yavuz, E., Turner, J., and Nowotny, T. (2016). GeNN: a code generation framework for accelerated brain simulations. *Sci. Rep.* 6, 18854. doi: 10.1038/srep18854.

Yik, J., Ahmed, S. H., Ahmed, Z., Anderson, B., Andreou, A. G., Bartolozzi, C., et al. (2023). NeuroBench: Advancing Neuromorphic Computing through Collaborative, Fair and Representative Benchmarking. *arXiv Prepr. arXiv2304.04640*.

Zenke, F., and Gerstner, W. (2014). Limits to high-speed simulations of spiking neural networks using general-purpose computers. *Front. Neuroinform.* 8, 76. doi: 10.3389/fninf.2014.00076.

Zimmer, R., Pellegrini, T., Singh, S. F., and Masquelier, T. (2019). Technical report: supervised training of convolutional spiking neural networks with PyTorch. *arXiv Prepr. arXiv1911.10124*.
